# Supplementary material for: Quality of maternal and newborn care in limited-resource settings: a facility-based cross-sectional study in Burkina Faso and Côte d’Ivoire
Source: BMJ Open. 2020 Jun 7;10(6):e036121. doi: 10.1136/bmjopen-2019-036121 (PMC7282325; doi:10.1136/bmjopen-2019-036121)
Supplement: Author's manuscript [file bmjopen-2019-036121.draft_revisions.pdf]

BMJ Open

BMJ Open is committed to open peer review. As part of this commitment we make the peer review history of every article we publish publicly available.

When an article is published we post the peer reviewers' comments and the authors' responses online. We also post the versions of the paper that were used during peer review. These are the versions that the peer review comments apply to.

The versions of the paper that follow are the versions that were submitted during the peer review process. They are not the versions of record or the final published versions. They should not be cited or distributed as the published version of this manuscript.

BMJ Open is an open access journal and the full, final, typeset and author-corrected version of record of the manuscript is available on our site with no access controls, subscription charges or pay-per-view fees (http://bmjopen.bmj.com).

If you have any questions on BMJ Open's open peer review process please email info.bmjopen@bmj.com

BMJ Open

Sub-optimal quality of maternal and newborn care in limited resources settings: a facility-based cross-sectional study in Burkina Faso and Côte d’Ivoire.

Journal:	*BMJ Open*
Manuscript ID	bmjopen-2019-036121
Article Type:	Original research
Date Submitted by the Author:	01-Dec-2019
Complete List of Authors:	Millogo, Tieba; African Institute of Public Health, Epidemiology; Institut de Recherche en Sciences de la Sante<br>Agbre-Yace , Marie; Institut National de Santé Publique (INSP-Côte d’Ivoire)<br>Kourouma, Raissa; Institut National de Santé Publique (INSP-Côte d’Ivoire)<br>Yaméogo, W. Maurice; Institut de Recherche en Sciences de la Sante<br>Tano-Kamelan , Akoua ; Institut National de Santé Publique (INSP-Côte d’Ivoire)<br>Sissoko, Fatou; Institut de Recherche en Sciences de la Sante<br>Soltié-Coulibaly, Aminata; Institut National de Santé Publique (INSP-Côte d’Ivoire)<br>Thorson, Anna; Human Reproduction Program/World Health Organization (Geneva)<br>Kouanda, Seni; Institut de Recherche en Sciences de la Sante; African Institute of Public Health
Keywords:	Quality in health care < HEALTH SERVICES ADMINISTRATION & MANAGEMENT, PRIMARY CARE, PUBLIC HEALTH

SCHOLARONE™
Manuscripts

I, the Submitting Author has the right to grant and does grant on behalf of all authors of the Work (as defined in the below author licence), an exclusive licence and/or a non-exclusive licence for contributions from authors who are: i) UK Crown employees; ii) where BMJ has agreed a CC-BY licence shall apply, and/or iii) in accordance with the terms applicable for US Federal Government officers or employees acting as part of their official duties; on a worldwide, perpetual, irrevocable, royalty-free basis to BMJ Publishing Group Ltd ("BMJ") its licensees and where the relevant Journal is co-owned by BMJ to the co-owners of the Journal, to publish the Work in this journal and any other BMJ products and to exploit all rights, as set out in our licence.

The Submitting Author accepts and understands that any supply made under these terms is made by BMJ to the Submitting Author unless you are acting as an employee on behalf of your employer or a postgraduate student of an affiliated institution which is paying any applicable article publishing charge ("APC") for Open Access articles. Where the Submitting Author wishes to make the Work available on an Open Access basis (and intends to pay the relevant APC), the terms of reuse of such Open Access shall be governed by a Creative Commons licence – details of these licences and which Creative Commons licence will apply to this Work are set out in our licence referred to above.

Other than as permitted in any relevant BMJ Author's Self Archiving Policies, I confirm this Work has not been accepted for publication elsewhere, is not being considered for publication elsewhere and does not duplicate material already published. I confirm all authors consent to publication of this Work and authorise the granting of this licence.

For peer review only

Sub-optimal quality of maternal and newborn care in limited resources settings: a facility-based cross-sectional study in Burkina Faso and Côte d'Ivoire.

Tieba Millogo : African Institute of Public Health (IASP) & Institut de Recherche en Sciences de la Santé, Burkina Faso. millogorod@gmail.com ; tmillogo@iasp-bf.org .

Marie Laurette Agbre-Yace : Institut National de Santé Publique (INSP-Côte d'Ivoire). agbreyace@yahoo.fr .

Raissa K Kourouma : Institut National de Santé Publique (INSP-Côte d'Ivoire). kkouroum@gmail.com .

Maurice W E Yaméogo : African Institute of Public Health (IASP) & Institut de Recherche en Sciences de la Santé, Burkina Faso. yamwamb@gmail.com .

Akoua Tano-Kamelan : Institut National de Santé Publique (INSP-Côte d'Ivoire). tanokamelan@yahoo.fr .

Fatou B Sissoko : Institut de Recherche en Science de la Santé (IRSS), Burkina Faso. bfatou.sissoko@yahoo.fr .

Aminata S Coulibaly-Koné : Institut National de Santé Publique (INSP-Côte d'Ivoire). soltieamy@yahoo.fr .

Anna Thorson : Human Reproduction Program/World Health Organization (Geneva). thorsona@who.int .

Seni Kouanda : Institut de Recherche en Sciences de la Santé, Burkina Faso & African Institute of Public Health (IASP). senikouanda@gmail.com .

Correspondence : **Tieba Millogo** : African Institute of Public Health. 12 BP 199 Ouagadougou. Burkina Faso. millogorod@gmail.com ; tmillogo@iasp-bf.org . Cel:+22671910013.

Abstract word count : 245
Manuscript word count: 3533

For peer review only

For peer review only - http://bmjopen.bmj.com/site/about/guidelines.xhtml

Abstract

Sub-optimal quality of maternal and newborn care in limited resources settings: a facility-based cross-sectional study in Burkina Faso and Côte d'Ivoire.

Abstract word count : 245

Manuscript word count: 3533

Objective: To assess and compare the quality of the intrapartum and immediate postpartum care in Burkina Faso and Côte d'Ivoire using validated process indicators.

Design : Health facility based cross-sectional study with a direct observation of health care wokers's practices while caring for mother-newborn pairs during the intrapartum and immediate postpartum periods.

Setting: Primary health care facilities and their corresponding referral hospitals in the Central-North region in Burkina Faso and the Agneby-Tiassa-Mé region in Côte d'Ivoire.

Participants: Health care providers who care for mother-newborn pairs during intrapartum and immediate postpartum periods, the labouring women and their newborns after childbirth.

Main Outcome Measure(s) : Adherence to Essential Best Practices (EBPs) at four pause-points in each birth event and the overall quality score based on the level of adherence to the set of EBPs observed for a selected pause-point.

Results: Overall the compliance with EBPs was sub-optimal at all the four pause-points, even though it varied widely from one EBP to another. The adherence was very low with respect to hands hygiene practices (<10% for wearing sterile gloves before vaginal examination in both countries) and was very high with respect to thermal management of newborns (>90% in both countries). The overall mean quality scores were consistently higher for all pause-points in Burkina Faso as compared to Côte d'Ivoire (p<0.001).

Conclusions: Women delivering in health care facilities do not always receive proven EBPs needed to prevent poor childbirth outcomes. There is a need for quality improvement interventions.

Key words: Quality maternal and newborn care, Burkina Faso, Côte d'Ivoire

Strengths and limitations of the study

- Random selection of health facilities in two different countries and observation of sufficient sample sizes of birth events in each country;
- Direct and prospective assessment of healthcare workers'adherence to EBPs around the time of childbirth by trained healthcare providers as opposed to retrospective ascertainement through medical records in many previous studies;
- Non-disclosing of the items being observed the healthcare providers whose care practices were being observed;
- Use of context specific validated EBPs to maximise relevance of the study findings.
- Because of the direct observation of the practices, we cannot rule out a Hawthorne effect and the data were collected by 8 data collectors in each country and are thus subject to an inter-rater reliability bias even though they were trained to standardize the processes.

For peer review only

For peer review only - http://bmjopen.bmj.com/site/about/guidelines.xhtml

Introduction

Reducing the high burden of preventable maternal and neonatal deaths remains an important health-related goal in the sustainable development era. Although tremendous progresses have been achieved globally during the last two decades, most of the highly affected countries have failed to meet the target of reducing by ¾ their 1990 maternal mortality ratios by 2015[1,2]. Around 830 women are still dying every day from pregnancy and childbirths related complications, of which 99% are occurring in low- and middle income countries and more than 50% in sub-Saharan Africa[1,3]. There is a strong evidence showing that the largest burden of maternal death is clustered around the time of childbirth and immediate post-partum[4]. A Global advocacy for skilled attendance at each birth, aiming to reduce preventable maternal deaths during the MDGs'era [5,6], resulted in a significant increase in the access and uptake of childbirth services globally including in sub-Saharan African countries [7,8]. This was however not paralleled by the expected reduction in maternal and infant mortality rates in low-resourced settings [9]. A skilled birth attendant and health facility delivery will improve maternal and newborn outcomes only if they encompass the delivery of proven effective interventions needed to prevent or to manage the major causes of maternal and newborn morbidity and mortality.

When the quality of care is sub-standard these principles are challenged [12]. The poor-quality of care in health facilities, specifically in low-resource settings, represents a neglected agenda[13] and is increasingly recognized as a major contributing factor to persistent high levels of preventable maternal and newborn morbidity and mortality [12]. Closing the gaps in quality of care will be central to the achievement of the global commitment towards Universal Health Coverage (UHC)[14]. One of the pillars of the UHC will be to make quality services available to individuals and communities to meet their health-related needs without experiencing financial hardships. The quality of care is however complex and encompasses various approaches and dimensions. Quality of maternal and newborn care is very often assessed through delivery outcomes or the availability of resources needed to deliver effective interventions[15–17], rarely using process indicators[18]. While childbirth outcomes are relevant indicators of quality of care, they do not provide information on the process of care and thus neither on quality gaps that need to be filled. Very few studies have investigated the quality of maternal and newborn care in SSA using process indicators. We intend in this study to assess and compare the quality of the intrapartum and immediate postpartum care in two SSA countries using established process indicators[18].

For peer review only - http://bmjopen.bmj.com/site/about/guidelines.xhtml

Methods

Study settings

Burkina Faso (low-income country) and Côte d'Ivoire (low-middle income country), two sub-Saharan African countries with maternal mortality ratios among the highest in the world, have both witnessed important increase in the uptake of childbirth services during the last two decades, with health facility births reaching nearly 90%[10]. Yet, both countries are struggling to achieve the desire reduction in maternal deaths and the current pace must be further accelerated if they are to meet the the target 3.8 of the SDGs of not having more than 140 maternal deaths per 100000 live births by 2030[11]. The study was carried out in two purposively selected regions, one in each country. In Burkina Faso, the Central-North health region hosted the study, while in Côte d'Ivoire it was conducted in the Agneby-Tiassa-Mé health region. Both regions have their capital cities located approximately 100 km away from the respective capital city of its country. The two regions have similar health care delivery systems with a regional hospital then, district hospitals each serving, a health district comprised of several primary health care centers. The central-north health region in Burkina Faso has got six health districts and 130 primary health care facilities (PHCF) and the Agnéby-Tiassa-Mé health region of Côte d'Ivoire is divided into seven health districts with 131 PHCF.

Study design

We used a facility-based cross-sectional study in government-own health facilities, consisting of a direct and passive observation of the health care providers' management of the delivery.

For peer review only - http://bmjopen.bmj.com/site/about/guidelines.xhtml

Study population, sampling and sample size calculation

We randomly selected five (5) health districts in each region. The district hospitals of the selected health districts along with the regional hospital were systematically included in the study in each country. Based on the average childbirth caseloads per levels of care (hospitals and PHCF) and a maximum length of facility stay of 5 days per data collectors' team, it was estimated that a minimum of 65 PHCFs would be needed in each country to achieve the calculated sample size. In the catchment area of each selected health district, up to 15 eligible PHCF were randomly selected to be included in the study. Eligible PHCFs were those with a minimum average caseload of 1.5 childbirths per day as reported in the health records of the same period in the previous year (during the last quarter of 2017).

The study population comprised :

- All the health care providers involved in intrapartum and immediate postpartum care, irrespective of their qualifications. They were offered to participate in the study and we excluded health care providers who were in the health facility for their qualifying internship.
- All the pregnant women in labour, admitted without c-section or referral needs identified at admission. We consecutively recruited all eligible pregnant women during the data collection period in each health facility. Exclusion criteria were <18 years in both countries, pregnant women seen at an advanced stage of the labor (third stage) with frequent (≤ 5mn) and intense uterine contractions that would prevent them to provide a valid informed consent.

The sample size of pregnant women was calculated using the conservative proportion of 50% adherence to each Essential Best Practice (EBP)with a precision of 5% and type I error of 5%. To account for the clustering nature of the data, the calculated sample size was adjusted using a design effect based on an intraclass correlation coefficient of 0.01 [19] and a size of 15

For peer review only - http://bmjopen.bmj.com/site/about/guidelines.xhtml

observations per group. The minimum sample size was estimated to be 425 pregnant women to be included in the observation per pause-point in each country, giving a minimum total number of 1700 of observations per country.

Data collection tools and processes

Data collectors with a medical background (medical students, midwives) and who had undergone a two-days training including a field testing of the study tools, were appointed in the delivery room to directly observe and document health care providers' adherence to EBPs at four critical points: admission, before the woman starts pushing, immediate postpartum (soon after birth to ≤ 1h) and before discharge from the health facility. The EBPs were derived from the World Health Organization Safe Childbirth Checklist (WHO-SCC) [20]complemented by a list of other validated quality indicators for SSA[18] (see table 1). A total of 34 EBPs were observed during each birth event. Adherence to each EBP was measured using a binary yes/no variable. The observation was strictly passive with minimum interaction between observer and health care workers and the patients. The data collectors were only allowed to intervene in the event of a life-threatening complication. The data collection took place from 12th November to 20th December 2018 and lasted for up to five (5) days in each health facility. Observations were conducted every day of the week and at any time without discontinuation, provided an eligible woman is present. A data collector could observe all or part of the four points for each birth event. However, data pertaining to the same critical juncture were fully collected by the same data collector. Thus, once a data collector had initiated the observation for a given pause points, he/she could get off work only after having filled in all the items pertaining to that pause point.

For peer review only - http://bmjopen.bmj.com/site/about/guidelines.xhtml

Table 1: Essential Best Practices (EBPs) to be observed for health workers' compliance at each pause-point

N°	Admission	Before pushing	Soon after birth	Before discharge
1	○ Washes his/her hands before examination	○ Sterile gloves available	○ Clamps or ties cord correctly	○ Assesses the mother for vaginal bleeding
2	○ Wears sterile gloves before vaginal examination	○ Soap and clean water available	○ Immediately dries the baby with clean towel	○ Assesses the baby for the quality of breastfeeding
3	○ Asks for vaginal bleedings	○ Prepares a prefilled syringe with uterotonics for AMTSL	○ Assesses for vaginal and perineal lacerations	○ Discusses postpartum family planning with the woman/couple
4	○ Asks for vision blurred	○ Clean towel available	○ Assesses for placenta completeness	○ Plans postpartum visits and makes sure they will consult in the event of a complication
5	○ Checks HIV status	○ Sterile blade or scissor available	○ Assesses new born for need of reference	
6	○ Measures blood pressure	○ Face mask available	○ Takes mother vital signs within 15 minutes after	
7	○ Takes pulse	○ Suction device available	○ Palpates uterus within 15 minutes after birth	
8	○ Uses partograph where indicated	○ Assistant birth attendant identified and ready to intervene-	○ Initiates breastfeeding within 1 hour	
9	○ Takes temperature-yes		○ Skin-to-skin contact initiated within 1 hour	
10	○ Assesses for need of referral		○ Informs companion/woman to call if complications	
11	○ Encourages companion to assist			
12	○ Informs companion/woman to call in case of complications			

For peer review only

For peer review only - http://bmjopen.bmj.com/site/about/guidelines.xhtml

Data analysis

Descriptive statistics were computed using means and standard deviations or medians and interquartile ranges (IQR) for numerical variables, and proportions were used to describe categorical variables. We calculated the level of adherence to each EBP as the proportion of childbirth events in which the EBP was effectively performed. For each pause-point, based on complete cases, a quality score was computed as a number of EBP that were effectively performed at each birth event. We further used two independent samples t-tests to compare mean quality scores at the four clinical junctures between the countries. Significance was considered if p-value <0.05.

Ethics

The research protocol was submitted and obtained ethics approvals from the national ethics review committees of Burkina Faso (reference number 2017-4-043) and Côte d'Ivoire (161-18/MSHP/CNE SVS-Kp). It was also approved by the ethics review committee of the WHO (protocol ID ERC.0002951). A written informed consent was obtained from all participants in the study. Data were collected, managed and analyzed in a way to ensure the confidentiality of study participants.

Patient and public involvement

There has been no patient and/or public involvement in the study design, data collection, data analysis and writing of this research.

Results

A total of 73 and 69 health facilities were respectively included in the study in Burkina Faso and Côte d'Ivoire. In total, 532 and 627 pregnant women in labour gave informed consent to be included in the observations for the pause-point I in Burkina Faso and Côte d'Ivoire

For peer review only - http://bmjopen.bmj.com/site/about/guidelines.xhtml

respectively. The included women that were not referred to another health facility or transferred in a different service were observed through the four pause-points if they were discharged from the health facility during the observation period. The numbers of birth events observed by pause-point by country and by health facility types are presented in table 2.

Table 2: Numbers of births events observed by pause-point by country and types of health facilities

Pause-points	*Burkina Faso*			*Côte d'Ivoire*		
	**Hospitals**	**PHCFs**	**Total**	**Hospitals**	**PHCFs**	**Total**
Admission	103	429	**532**	114	513	**627**
Before pushing	98	418	**516**	108	478	**586**
Soon after delivery	98	415	**513**	108	477	**585**
Before discharge	92	411	**503**	105	474	**579**
**Number of observations**	**391**	**1673**	**2064**	**435**	**1942**	**2377**

At the admission, compliance with EBPs ranged from 8.3% (wears sterile gloves for vaginal examination) to 99.4% (assesses the woman for need of referral) in Burkina Faso. In Côte d'Ivoire, the compliance ranged from 5.5% (wears sterile gloves for vaginal examination) to 84.7% (assesses the woman for need of referral).

During the immediate pre-pushing phase, evidence-best practices were implemented in 45.2% (assistant birth attendant available and ready to help) to 97.3% (sterile gloves available) of deliveries in Burkina Faso and from 6.8% (prepares face mask for neonatal resuscitation) to 95.9% (sterile gloves available) in Côte d'Ivoire.

Soon after birth, the compliance ranged from 52.1% (takes vital signs within 15mn) to 99.4% (immediately dries the baby with clean towels) in Burkina Faso and from 19.4% (clamps or ties correctly the cord) to 97.6% (assesses for vaginal or perineal lacerations) in Côte d'Ivoire.

When leaving the facility , 47.2% of women in Burkina Faso were offered a discussion on postpartum family planning and in Cote d'Ivoire the lowest adherence to best practices was for assessment of quality of breastfeeding (23.3%). In both countries close to all women were assessed for vaginal bleeding.

For peer review only - http://bmjopen.bmj.com/site/about/guidelines.xhtml

The levels of adherence to each evidence based practice per stage and per country is presented in table 3.

Table 3: Compliance to EBPs by pause-point and by country.

Essential best practices	Burkina Faso n (%)	Côte d'Ivoire n (%)
**Admission**	(N=532)	(N=627)
Washes his/her hands before examination	106 (20.4)	34 (5.5)
Wears sterile gloves before vaginal examination	44 (8.3)	55 (9.0)
Asks for vaginal bleedings	236 (44.9)	167 (27.0)
Asks for vision blurred	213 (40.5)	150 (24.3)
Checks HIV status	422 (79.8)	417 (68.4)
Measures blood pressure	399 (75.1)	484 (77.7)
Takes pulse	224 (42.3)	265 (42.7)
Uses partograph where indicated	176 (48.9)	114 (23.1)
Takes temperature	356 (66.8)	236 (37.6)
Assesses for need of referral	529 (99.4)	530 (84.7)
Encourages companion to assist to the childbirth	202 (38.5)	54 (9.3)
Informs companion/woman to call in case of complications	463 (87.7)	322 (55.8)
**Before pushing or before caesarian section**		
Sterile gloves available	501 (97.3)	562 (95.9)
Soap and clean water available	327 (63.5)	448 (76.5)
Prepares a prefilled syringe with uterotonics for AMTSL	440 (85.4)	441 (75.3)
Clean towel available	485 (95.2)	518 (88.4)
Sterile blade or scissor available	498 (96.7)	472 (80.5)
Face mask available	244 (47.4)	40 (6.8)
Suction device available	392 (76.1)	159 (27.1)
Assistant birth attendant identified and ready to intervene	233 (45.2)	75 (12.8)
**Soon after birth (within one hour)**		
Clamps or ties cord correctly	316 (61.5)	114 (19.4)
Immediately dries the baby with clean towel	511 (99.4)	544 (92.8)
Assesses for vaginal and perineal lacerations	488 (94.9)	572 (97.6)
Assesses for placenta completeness	280 (54.6)	204 (34.9)
Assesses new born for need of reference	509 (99)	496 (84.8)
Takes mother vital signs within 15 minutes after	268 (52.1)	272 (46.5)
Palpates uterus within 15 minutes after birth	416 (80.9)	334 (57.1)
Initiates breastfeeding within 1 hour	361 (74)	212 (39.7)
Skin-to-skin contact initiated within 1 hour	405 (80.5)	309 (54.3)
Informs companion/woman to call if complications	445 (86.6)	439 (75)
**Before discharge from the health facility**		
Assesses the mother for vaginal bleeding	365 (72.7)	374 (64.6)
Assesses the baby for the quality of breastfeeding	276 (55.1)	135 (23.3)
Discusses postpartum family planning with the woman/couple	237 (47.2)	204 (35.2)
Plans postpartum visits and informs to consult if complications	435 (86.7)	453 (78.1)

For peer review only - http://bmjopen.bmj.com/site/about/guidelines.xhtml

The overall mean quality scores were consistently higher for all pause-points in Burkina Faso as compared to Côte d'Ivoire (p<0.001). The distribution of the scores of quality by pause-point and by country is depicted in figure1.

Discussion

In this study we assessed health care providers' adherence to evidence based practices (EBPs) in delivery care using direct observation. Our results showed a wide diversity in adherence to EBPs ranging from very low to very high implementation. Overall, the adherence to EBPs was better in Burkina Faso as compared to Côte d'Ivoire. Though important quality gaps were shown in both countries. Hand hygiene (washing hands before examination and wearing sterile gloves for each vaginal examination) was very poor in both countries despite the widespread availability of hand hygiene facilities in both countries (results not presented). With equipment available, adherence to hygiene routines is a fundamental and non-demanding practice for the prevention of infections [21,22]. Yet, non-compliance is widespread and remains a source of concern globally [23,24].

The history taking of critical information such as history of vaginal bleedings or vision blurred during the pregnancy was sub-optimal in Burkina Faso and poor in Côte d'Ivoire. Omission of measuring blood pressure was common, and occurred in 25% of women In Côte d'Ivoire. temperature was not measured in 2/3 of women in labour, missing an opportunity to identify infections.

These gaps in initial assessment can cause at risk patients or prevailing complications to go undetected. It is also noteworthy that, despite being an ancient and for long systematized intervention, a partograph to monitor labor was used during less than half of thedeliveries in both countries. The low-utilization of the partograph and its poor quality when used, are well recognized and have been widely reported in the literature[25–27].

The assessment of vaginal bleedings and perineal lacerations and the preparation of a prefilled syringe with uterotonics for the active management of the third stage of labor (AMTSL) were satisfactory adhered to in both countries (>75%). These are important practices for the prevention, early detection and management of postpartum hemorrhage (PPH), a leading cause of maternal death[4]. The bedside availability of the prepared oxytocin improves its timely administration[28]. The good level of performance of these standards may be the result

For peer review only - http://bmjopen.bmj.com/site/about/guidelines.xhtml

of the rising awareness of the importance of PPH as a leading cause of death and the emphasis put on its prevention and management in both initial and refreshing trainings for health care providers.

Implementation of other practices equally relevant to PPH: the performance of “assessment of the completeness of the placenta” and “the monitoring of maternal vital signs” was however very low, and only provided to around half of the women in Burkina Faso for both EBPs, and to 1/3 and around 3/7 women in Côte d’Ivoire respectively. These are life-saving practices that can be implemented at low-cost, but which requires health-worker awareness and knowledge on the purpose and rationale for providing these services, in addition to clinical skills in executing them. The low proportion of women in both countries, that received these interventions, represent unmet needs of urgent action to address these cost-effective practices. Our findings also highlight the complexity of the peri- and immediate post-partum care, where a series of equally important steps need to be taken to minimize risks of maternal morbidity, and how failure to address all of this puts women at risk of severe consequences.

Thermal management of newborns was satisfactory adhered to in both countries through the availability of clean towels before the woman starts pushing and immediately drying the newborn soon after birth. The compliance with other lifesaving interventions for newborns (skin-to-skin contact, early initiation of breastfeeding) was beyond ¾ women in Burkina Faso and barely ½ women in Côte d’Ivoire.

Overall, supportive services (encouraging the companion to assist to the delivery, identifying and preparing an assistant birth attendant ready to help), at any point in time tend to be less adhered to as compared to other standards of care.

The health facilities in both countries rarely used the opportunity to provide post-partum contraceptive advice to the women attending services. Given the large unmet needs of modern contraceptives in both countries, implementation of post-partum family planning programmes is an important strategy to close the gap. Knowledge translation and follow-up of patients between health care workers engaged in intra-partum care and family planning is needed.

The same was previously reported[19] and is suggestive of the health workers propensity to give precedence to interventions needed to prevent or treat imminent and tangible complications. Noticeably, an important proportion of women and or companions received

For peer review only - http://bmjopen.bmj.com/site/about/guidelines.xhtml

information related to potential complications and signs of danger and what to do in case they occur in both countries from admission to discharge.

Study Limitations

In interpreting our results, some limitations should be kept in mind. We carried out a direct observation of facility based deliveries in two selected regions and the findings may not be generalizable to each individual country. Although we did not provide information to health care providers on the items being observed, we cannot rule out a Hawthorne effect and some may have changed their health care practices during the study period. The data collection was carried out by 8 data collectors in each country. Although they were trained by the same research team to standardized processes and judgements, we cannot also rule out issues related to inter-rater reliability. Finally, the non-compliance to a given standard does not always imply that the observed health care provider failed to do so. It could also be in some cases that the supplies needed to administer the intervention were not available.

Conclusion

Overall, the compliance to evidence based practices during delivery was insufficient and important quality of care gaps were identified. A significant number of women in Burkina Faso and Cote d'Ivoire do miss out on important interventions, especially related to infection prevention and control and prevention of post-partum hemorrhage. It is also noticeable that there are lost opportunities in relation to implementation of post-partum contraceptive strategies. Conditional on the availability of the supplies, the investigated EBPs are relatively low cost and easy to implement. Quality improvement interventions in the form of reminders tools for health care providers, such as the WHO safe childbirth check-list have the potential to sustain currently performed best practices while filling the gaps where they persist.

Authors' contributions

All authors meet the criteria recommended by the International Committee of Medical Journal Editors (ICMJE). All authors made substantial contributions to conception and design, acquisition of data or analysis and interpretation of data. SK,MT,MLA conceived the study design. MT analyzed the data and proposed the first draft of the paper. SK, MLA, RKK,

For peer review only - http://bmjopen.bmj.com/site/about/guidelines.xhtml

MWY, AT, FBS, ASC and AT revised and contributed to the successive drafts. The final version of the paper was revised and approved by all the authors.

Acknowledgment

We are grateful to WHO Human Reproduction Program for funding the research. The funders have no role in the design of the research, nor they have in the collection, analysis and interpretation of the data.

Data availability statement

The data presented in this study are from the Checklist study conducted in Burkina Faso and Côte d'Ivoire. Data can be accessed upon request sent to the principal investigatior of the study, Prof Seni Kouanda at senikouanda@gmail.com. Access will be granted only after careful and due consideration of the compliance with the ethics requirements and the data policy of the Institut de Recherche en Sciences de la Santé.

Funding sources

The study was funded by WHO/HRP.

Competing interests

All authors have declared no competing interests.

For peer review only

For peer review only - http://bmjopen.bmj.com/site/about/guidelines.xhtml

References

1. Alkema L, Chou D, Hogan D, Zhang S, Moller A-B, Gemmill A, Fat DM, Boerma T, Temmerman M, Mathers C, Say L. Global, regional, and national levels and trends in maternal mortality between 1990 and 2015, with scenario-based projections to 2030: a systematic analysis by the UN Maternal Mortality Estimation Inter-Agency Group. The Lancet. 2016 Jan;387(10017):462–74.
2. Victora CG, Requejo JH, Barros AJD, Berman P, Bhutta Z, Boerma T, Chopra M, de Francisco A, Daelmans B, Hazel E, Lawn J, Maliqi B, Newby H, Bryce J. Countdown to 2015: a decade of tracking progress for maternal, newborn, and child survival. The Lancet. 2016 May;387(10032):2049–59.
3. World Health Organization, UNICEF, United Nations, Department of Economic and Social Affairs, Population Division, World Bank. Trends in maternal mortality: 1990 to 2015 : estimates by WHO, UNICEF, UNFPA, World Bank Group and the United Nations Population Division [Internet]. 2015 [cited 2017 Dec 17]. Available from: http://www.who.int/reproductivehealth/publications/monitoring/maternal-mortality-2015/en/
4. Ronsmans C, Graham WJ, Lancet Maternal Survival Series steering group. Maternal mortality: who, when, where, and why. Lancet. 2006 Sep 30;368(9542):1189–200.
5. Bullough C, Meda N, Makowiecka K, Ronsmans C, Achadi EL, Hussein J. REVIEW: Current strategies for the reduction of maternal mortality: REVIEW. BJOG Int J Obstet Gynaecol. 2005 Jun 29;112(9):1180–8.
6. World Health Organization. Making pregnancy safer the critical role of the skilled attendant: a joint statement by WHO, ICM and FIGO. Geneva: World health organization; 2004.
7. Udo IE, Doctor HV. Trends in Health Facility Births in sub-Saharan Africa: An Analysis of Lessons Learned under the Millennium Development Goal Framework. Afr J Reprod Health [Internet]. 2017 May 22 [cited 2017 Dec 18];20(3). Available from: https://www.ajrh.info/index.php/ajrh/article/view/175
8. Doctor HV, Nkhana-Salimu S, Abdulsalam-Anibilowo M. Health facility delivery in sub-Saharan Africa: successes, challenges, and implications for the 2030 development agenda. BMC Public Health. 2018 Dec;18(1):765.

For peer review only - http://bmjopen.bmj.com/site/about/guidelines.xhtml

9. Ekirapa-Kiracho E, Waiswa P, Rahman MH, Makumbi F, Kiwanuka N, Okui O, Rutebemberwa E, Bua J, Mutebi A, Nalwadda G, Serwadda D, Pariyo GW, Peters DH. Increasing access to institutional deliveries using demand and supply side incentives: early results from a quasi-experimental study. BMC Int Health Hum Rights. 2011;11 Suppl 1:S11.

10. Ministère de la Santé. Annuaire Statistique 2015 [Internet]. Ouagadougou; 2016 Mar. Available from: http://www.sante.gov.bf/index.php?option=com_edocman&task=document.viewdoc&id=113&Itemid=1123

11. Alkema L, Chou D, Hogan D, Zhang S, Moller A-B, Gemmill A, Fat DM, Boerma T, Temmerman M, Mathers C, Say L. Global, regional, and national levels and trends in maternal mortality between 1990 and 2015, with scenario-based projections to 2030: a systematic analysis by the UN Maternal Mortality Estimation Inter-Agency Group. The Lancet. 2016 Jan;387(10017):462–74.

12. Kongnyuy EJ, Mlava G, van den Broek N. Facility-Based Maternal Death Review In Three Districts In The Central Region of Malawi. Womens Health Issues. 2009 Jan;19(1):14–20.

13. van den Broek N, Graham W. Quality of care for maternal and newborn health: the neglected agenda: Quality of care for maternal and newborn health. BJOG Int J Obstet Gynaecol. 2009 Oct;116:18–21.

14. Chan M, Director-General W. QUALITY OF CARE IN THE CONTEXT OF UNIVERSAL HEALTH COVERAGE (UHC). :2.

15. Admasu K, Haile-Mariam A, Bailey P. Indicators for availability, utilization, and quality of emergency obstetric care in Ethiopia, 2008. Int J Gynecol Obstet. 2011 Oct;115(1):101–5.

16. Wilunda C, Oyerinde K, Putoto G, Lochoro P, Dall'Oglio G, Manenti F, Segafredo G, Atzori A, Criel B, Panza A, Quaglio G. Availability, utilisation and quality of maternal and neonatal health care services in Karamoja region, Uganda: a health facility-based survey. Reprod Health [Internet]. 2015 Dec [cited 2017 Dec 18];12(1). Available from: http://reproductive-health-journal.biomedcentral.com/articles/10.1186/s12978-015-0018-7

17. Baguiya A, Meda IB, Millogo T, Kourouma M, Mouniri H, Kouanda S. Availability and utilization of obstetric and newborn care in Guinea: A national needs assessment. Int J Gynaecol Obstet Off Organ Int Fed Gynaecol Obstet. 2016 Nov;135 Suppl 1:S2–6.

18. Tripathi V, Stanton C, Strobino D, Bartlett L. Development and Validation of an Index

For peer review only - http://bmjopen.bmj.com/site/about/guidelines.xhtml

to Measure the Quality of Facility-Based Labor and Delivery Care Processes in Sub-Saharan Africa. Duan T, editor. PLOS ONE. 2015 Jun 24;10(6):e0129491.

19. Kumar S, Yadav V, Balasubramaniam S, Jain Y, Joshi CS, Saran K, Sood B. Effectiveness of the WHO SCC on improving adherence to essential practices during childbirth, in resource constrained settings. BMC Pregnancy Childbirth [Internet]. 2016 Dec [cited 2017 Jan 17];16(1). Available from: http://bmcpregnancychildbirth.biomedcentral.com/articles/10.1186/s12884-016-1139-x

20. Spector JM, Lashoher A, Agrawal P, Lemer C, Dziekan G, Bahl R, Mathai M, Merialdi M, Berry W, Gawande AA. Designing the WHO Safe Childbirth Checklist program to improve quality of care at childbirth. Int J Gynecol Obstet. 2013 Aug;122(2):164–8.

21. Allegranzi B, Pittet D. Role of hand hygiene in healthcare-associated infection prevention. J Hosp Infect. 2009 Dec;73(4):305–15.

22. Pittet D, Allegranzi B, Storr J. The WHO Clean Care is Safer Care programme: Field-testing to enhance sustainability and spread of hand hygiene improvements. J Infect Public Health. 2008;1(1):4–10.

23. Musuuza JS, Barker A, Ngam C, Vellardita L, Safdar N. Assessment of Fidelity in Interventions to Improve Hand Hygiene of Healthcare Workers: A Systematic Review. Infect Control Hosp Epidemiol. 2016 May;37(5):567–75.

24. Anargh V, Singh H, Kulkarni A, Kotwal A, Mahen A. Hand hygiene practices among health care workers (HCWs) in a tertiary care facility in Pune. Med J Armed Forces India. 2013 Jan;69(1):54–6.

25. Asibong U, Okokon IB, Agan TU, Oku A, Opiah M, Essien EJ, Monjok E. The use of the partograph in labor monitoring: a cross-sectional study among obstetric caregivers in General Hospital, Calabar, Cross River State, Nigeria. Int J Womens Health. 2014;6:873–80.

26. Opiah MM, Ofi AB, Essien EJ, Monjok E. Knowledge and Utilization of the Partograph among Midwives in the Niger Delta Region of Nigeria. Afr J Reprod Health Rev Afr Santé Reprod. 2012;16(1):125–32.

27. Mandiwa C, Zamawe C. Documentation of the partograph in assessing the progress of labour by health care providers in Malawi's South-West zone. Reprod Health. 2017 Dec;14(1):134.

28. Moucheraud C, Gass J, Lipsitz S, Spector J, Agrawal P, Hirschhorn LR, Gawande A, Kodkany B. Bedside Availability of Prepared Oxytocin and Rapid Administration After

For peer review only - http://bmjopen.bmj.com/site/about/guidelines.xhtml

Delivery to Prevent Postpartum Hemorrhage: An Observational Study in Karnataka, India. Glob Health Sci Pract. 2015 Jun;3(2):300–4.

For peer review only

Figure legends

Figure 1: The boxplots in figure 1 depict the overall scores of quality of care computed for each pause-point by country. The maximum quality score for any pause-point equates to the number EBPs observed at that pause-point.

For peer review only

For peer review only - http://bmjopen.bmj.com/site/about/guidelines.xhtml

Figure legends

Figure 1: The boxplots in figure 1 depict the overall scores of quality of care computed for each pause-point by country. The maximum quality score for any pause-point equates to the number EBPs observed at that pause-point.

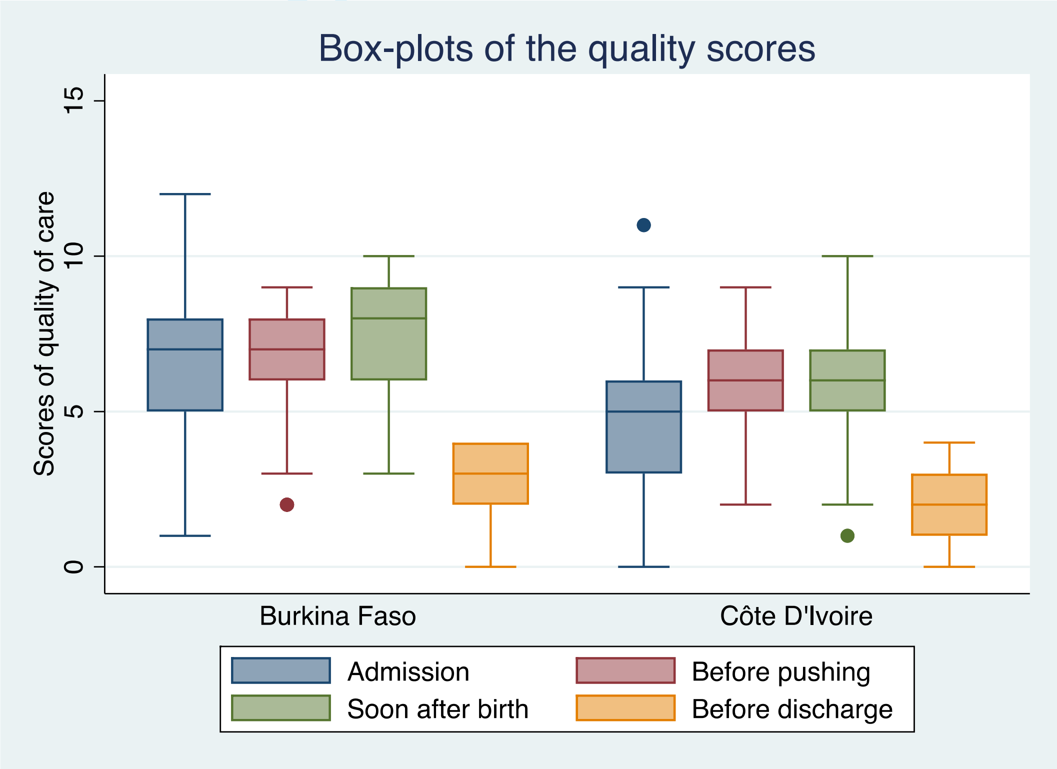

Figure 1: Distribution of quality scores per country and pause-points.

STROBE Statement—Checklist of items that should be included in reports of ***cross-sectional studies***

	Item No	Recommendation	Page No
**Title and abstract**	1	(*a*) Indicate the study's design with a commonly used term in the title or the abstract	1 &2
		(*b*) Provide in the abstract an informative and balanced summary of what was done and what was found	2
**Introduction**			
Background/rationale	2	Explain the scientific background and rationale for the investigation being reported	4
Objectives	3	State specific objectives, including any prespecified hypotheses	4
**Methods**			
Study design	4	Present key elements of study design early in the paper	5
Setting	5	Describe the setting, locations, and relevant dates, including periods of recruitment, exposure, follow-up, and data collection	5, 7
Participants	6	(*a*) Give the eligibility criteria, and the sources and methods of selection of participants	6
Variables	7	Clearly define all outcomes, exposures, predictors, potential confounders, and effect modifiers. Give diagnostic criteria, if applicable	8
Data sources/ measurement	8*	For each variable of interest, give sources of data and details of methods of assessment (measurement). Describe comparability of assessment methods if there is more than one group	*7*
Bias	9	Describe any efforts to address potential sources of bias	7
Study size	10	Explain how the study size was arrived at	6 &7
Quantitative variables	11	Explain how quantitative variables were handled in the analyses. If applicable, describe which groupings were chosen and why	9
Statistical methods	12	(*a*) Describe all statistical methods, including those used to control for confounding	9
		(*b*) Describe any methods used to examine subgroups and interactions	NA
		(*c*) Explain how missing data were addressed	9
		(*d*) If applicable, describe analytical methods taking account of sampling strategy	
		(*e*) Describe any sensitivity analyses	
**Results**			
Participants	13*	(a) Report numbers of individuals at each stage of study—eg numbers potentially eligible, examined for eligibility, confirmed eligible, included in the study, completing follow-up, and analysed	9&10
		(b) Give reasons for non-participation at each stage	10
		(c) Consider use of a flow diagram	
Descriptive data	14*	(a) Give characteristics of study participants (eg demographic, clinical, social) and information on exposures and potential confounders	10
		(b) Indicate number of participants with missing data for each variable of interest	
Outcome data	15*	Report numbers of outcome events or summary measures	10 & 11

For peer review only

Main results	16	(*a*) Give unadjusted estimates and, if applicable, confounder-adjusted estimates and their precision (eg, 95% confidence interval). Make clear which confounders were adjusted for and why they were included	12
		(*b*) Report category boundaries when continuous variables were categorized	
		(*c*) If relevant, consider translating estimates of relative risk into absolute risk for a meaningful time period	
Other analyses	17	Report other analyses done—eg analyses of subgroups and interactions, and sensitivity analyses	
**Discussion**			
Key results	18	Summarise key results with reference to study objectives	12
Limitations	19	Discuss limitations of the study, taking into account sources of potential bias or imprecision. Discuss both direction and magnitude of any potential bias	14
Interpretation	20	Give a cautious overall interpretation of results considering objectives, limitations, multiplicity of analyses, results from similar studies, and other relevant evidence	12 & 13
Generalisability	21	Discuss the generalisability (external validity) of the study results	14
**Other information**			
Funding	22	Give the source of funding and the role of the funders for the present study and, if applicable, for the original study on which the present article is based	15

*Give information separately for exposed and unexposed groups.

Note: An Explanation and Elaboration article discusses each checklist item and gives methodological background and published examples of transparent reporting. The STROBE checklist is best used in conjunction with this article (freely available on the Web sites of PLoS Medicine at http://www.plosmedicine.org/, Annals of Internal Medicine at http://www.annals.org/, and Epidemiology at http://www.epidem.com/). Information on the STROBE Initiative is available at www.strobe-statement.org.

For peer review only

Quality of maternal and newborn care in limited-resource settings: a facility-based cross-sectional study in Burkina Faso and Côte d’Ivoire

Journal:	*BMJ Open*
Manuscript ID	bmjopen-2019-036121.R1
Article Type:	Original research
Date Submitted by the Author:	11-Mar-2020
Complete List of Authors:	Millogo, Tieba; African Institute of Public Health, Epidemiology; Institut de Recherche en Sciences de la Sante<br>Agbre-Yace , Marie; Institut National de Santé Publique (INSP-Côte d’Ivoire)<br>Kourouma, Raissa; Institut National de Santé Publique (INSP-Côte d’Ivoire)<br>Yaméogo, W. Maurice; Institut de Recherche en Sciences de la Sante<br>Tano-Kamelan , Akoua ; Institut National de Santé Publique (INSP-Côte d’Ivoire)<br>Sissoko, Fatou; Institut de Recherche en Sciences de la Sante<br>Soltié-Coulibaly, Aminata; Institut National de Santé Publique (INSP-Côte d’Ivoire)<br>Thorson, Anna; Human Reproduction Program/World Health Organization (Geneva)<br>Kouanda, Seni; Institut de Recherche en Sciences de la Sante; African Institute of Public Health
**Primary Subject Heading**:	Public health
Secondary Subject Heading:	Health services research, Public health, Evidence based practice
Keywords:	Quality in health care < HEALTH SERVICES ADMINISTRATION & MANAGEMENT, PRIMARY CARE, PUBLIC HEALTH

I, the Submitting Author has the right to grant and does grant on behalf of all authors of the Work (as defined in the below author licence), an exclusive licence and/or a non-exclusive licence for contributions from authors who are: i) UK Crown employees; ii) where BMJ has agreed a CC-BY licence shall apply, and/or iii) in accordance with the terms applicable for US Federal Government officers or employees acting as part of their official duties; on a worldwide, perpetual, irrevocable, royalty-free basis to BMJ Publishing Group Ltd ("BMJ") its licensees and where the relevant Journal is co-owned by BMJ to the co-owners of the Journal, to publish the Work in this journal and any other BMJ products and to exploit all rights, as set out in our licence.

The Submitting Author accepts and understands that any supply made under these terms is made by BMJ to the Submitting Author unless you are acting as an employee on behalf of your employer or a postgraduate student of an affiliated institution which is paying any applicable article publishing charge ("APC") for Open Access articles. Where the Submitting Author wishes to make the Work available on an Open Access basis (and intends to pay the relevant APC), the terms of reuse of such Open Access shall be governed by a Creative Commons licence – details of these licences and which Creative Commons licence will apply to this Work are set out in our licence referred to above.

Other than as permitted in any relevant BMJ Author's Self Archiving Policies, I confirm this Work has not been accepted for publication elsewhere, is not being considered for publication elsewhere and does not duplicate material already published. I confirm all authors consent to publication of this Work and authorise the granting of this licence.

For peer review only

Quality of maternal and newborn care in limited-resource settings: a facility-based cross-sectional study in Burkina Faso and Côte d'Ivoire

Tieba Millogo : African Institute of Public Health (IASP) & Institut de Recherche en Sciences de la Santé, Burkina Faso. millogorod@gmail.com ; tmillogo@iasp-bf.org .

Marie Laurette Agbre-Yace : Institut National de Santé Publique (INSP-Côte d'Ivoire). agbreyace@yahoo.fr .

Raissa K Kourouma : Institut National de Santé Publique (INSP-Côte d'Ivoire). kkouroum@gmail.com .

W. Maurice E Yaméogo : African Institute of Public Health (IASP) & Institut de Recherche en Sciences de la Santé, Burkina Faso. yamwamb@gmail.com .

Akoua Tano-Kamelan : Institut National de Santé Publique (INSP-Côte d'Ivoire). tanokamelan@yahoo.fr .

Fatou B Sissoko : Institut de Recherche en Science de la Santé (IRSS), Burkina Faso. bfatou.sissoko@yahoo.fr .

Aminata Soltié-Coulibaly : Institut National de Santé Publique (INSP-Côte d'Ivoire). soltieamy@yahoo.fr .

Anna Thorson : Human Reproduction Program/World Health Organization (Geneva). thorsona@who.int .

Seni Kouanda : Institut de Recherche en Sciences de la Santé, Burkina Faso & African Institute of Public Health (IASP). senikouanda@gmail.com .

Correspondence : **Tieba Millogo** : African Institute of Public Health. 12 BP 199 Ouagadougou. Burkina Faso. millogorod@gmail.com ; tmillogo@iasp-bf.org . Cel:+22671910013.

Abstract word count : 271
Manuscript word count: 3735

For peer review only

ABSTRACT

Objective: To assess and compare the quality of intra-partum and immediate post-partum care across levels of health care in Burkina Faso and Côte d'Ivoire using validated process indicators.

Design: Health-facility-based cross-sectional study with direct observation of healthcare workers' practices while caring for mother–newborn pairs during intra-partum and immediate post-partum periods.

Setting: Primary healthcare facilities and their corresponding referral hospitals in the Central-North region in Burkina Faso and the Agneby-Tiassa-Mé region in Côte d'Ivoire.

Participants: Healthcare providers who care for mother–newborn pairs during intra-partum and immediate post-partum periods, the labouring women, and their newborns after childbirth.

Main Outcome Measure(s): Adherence to essential best practices (EBPs) at four pause-points in each birth event and the overall quality score based on the level of adherence to the set of EBPs observed for a selected pause-point.

Results: Overall, the compliance with EBPs was insufficient at all the four pause-points, even though it varied widely from one EBP to another. The adherence was very low with respect to hand hygiene practices: the care provider wore sterile gloves for vaginal examination in only 7.96% cases (95% confidence interval (CI): 5.66–11.06) in Burkina Faso and the care provider washed hands before examination in 6.71% cases (95% CI: 3.94–11.20) in Côte d'Ivoire. The adherence was very high with respect to thermal management of newborns in both countries (>90%). The overall mean quality scores were consistently higher in referral hospitals in Burkina Faso at all pause points excluding immediate post-partum.

For peer review only - http://bmjopen.bmj.com/site/about/guidelines.xhtml

Conclusions: Women delivering in healthcare facilities do not always receive proven EBPs needed to prevent poor childbirth outcomes. There is a need for quality improvement interventions.

Keywords: Quality maternal and newborn care, Burkina Faso, Côte d'Ivoire

Strengths and limitations of the study

- Random selection of health facilities in two different countries and observation of sufficient sample sizes of birth events in each country;
- Direct and prospective assessment of healthcare workers' adherence to essential best practices (EBPs) during and after childbirth by trained healthcare providers as opposed to retrospective ascertainment through medical records in many previous studies
- Use of context-specific validated EBPs to maximise relevance of the study findings
- Each country data are not representative of the whole country
- Because of the direct observation of the practices, we cannot rule out the Hawthorne effect and the data were collected by eight data collectors in each country and are thus subject to an inter-rater reliability bias even though they were trained to standardize the processes.

For peer review only

For peer review only - http://bmjopen.bmj.com/site/about/guidelines.xhtml

INTRODUCTION

Reducing the high burden of preventable maternal and neonatal deaths remains an important health-related goal in the sustainable development era. Although tremendous progress has been achieved globally in this area during the last two decades, most of the highly affected countries have failed to meet the target of reducing their 1990 maternal mortality ratios by 75% by 2015.[1,2] Around 830 women are still dying every day from pregnancy and childbirth-related complications, of which 99% are occurring in low- and middle-income countries and more than 50% in sub-Saharan Africa (SSA).[1,3] There is strong evidence showing that the largest burden of maternal death is clustered around the time of childbirth and immediate post-partum period.[4] A global advocacy for skilled attendance at each birth, aiming to reduce preventable maternal deaths during the Millennium Development Goals (MDGs) era,[5,6] resulted in a significant increase in the access and uptake of childbirth services globally, including in SSA countries.[7,8] This was however not paralleled by the expected reduction in maternal and infant mortality rates in low-resource settings.[9] A skilled birth attendant and health facility delivery will improve maternal and newborn outcomes only if they encompass the implementation of proven effective interventions needed to prevent or manage major causes of maternal and newborn morbidity and mortality.

When the quality of care is substandard, these principles are challenged.[10] The poor quality of care in health facilities, specifically in low-resource settings, represents a neglected agenda[11] and is increasingly recognized as a major contributing factor to persistent high levels of preventable maternal and newborn morbidity and mortality.[10] Closing the gaps in quality of care will be central to the achievement of the global commitment towards Universal Health Coverage (UHC).[12] One of the pillars of the UHC will be to make quality services available to individuals and communities to meet their health-related needs without experiencing financial hardships. Quality of care is however complex and encompasses

For peer review only

various approaches and dimensions. Quality of maternal and newborn care is very often assessed through delivery outcomes or the availability of resources needed to implement effective interventions,[13–15] rarely using process indicators.[16] While childbirth outcomes are relevant indicators of quality of care, they do not provide information on the process of care and, therefore, neither on quality gaps that need to be filled. Very few studies have investigated the quality of maternal and newborn care in SSA using process indicators. In this study, we intend to assess and compare the quality of intra-partum and immediate post-partum care in two SSA countries using established process indicators.[16]

METHODS

Study settings

Burkina Faso (low-income country) and Côte d'Ivoire (low-middle income country), two SSA countries with maternal mortality ratios among the highest in the world (310 and 614 per 100000 live births respectively),[17,18] have both witnessed significant increase in the uptake of childbirth services during the last two decades, with health facility births reaching nearly 90%.[19] Yet, both countries are struggling to achieve the desire reduction in maternal deaths and the current pace must be further accelerated if they are to meet Target 3.8 of the Sustainable Development Goals of not having more than 140 maternal deaths per 100000 live births by 2030.[20] The study was carried out in two regions where the collaborating research centres have locations, one in each country. In Burkina Faso, the Central-North health region hosted the study, while in Côte d'Ivoire, it was conducted in the Agneby-Tiassa-Mé health region. Both regions have their capital cities located approximately 100 km away from the respective capital city of its country. The two regions have similar healthcare delivery systems with a regional hospital, followed by district hospitals, each serving a health district comprised of several primary healthcare centres (PHCFs). The central-north health region in

For peer review only - http://bmjopen.bmj.com/site/about/guidelines.xhtml

Burkina Faso has six health districts and 130 primary healthcare facilities (PHCFs) and the Agnéby-Tiassa-Mé health region of Côte d'Ivoire is divided into seven health districts with 131 PHCFs.

Study design

We used a facility-based cross-sectional study in government-owned health facilities, enabling a direct and passive observation of the healthcare providers' management of deliveries.

Study population, sampling, and sample size calculation

In each selected region, we randomly selected five health districts. The district hospitals of the selected health districts along with the regional hospital were systematically included in the study in each country. Based on the average childbirth caseloads per level of care (hospital and PHCF) and a maximum length of facility stay of 5 days per data collector's team, it was estimated that a minimum of 65 PHCFs would be needed in each country to achieve the calculated sample size. In the catchment area of each selected health district, up to 15 eligible PHCFs were randomly selected to be included in the study. Eligible PHCFs were those with a minimum average caseload of 1.5 childbirths per day as reported in the health records of the same period in the previous year (during the last quarter of 2017).

The study population comprised

- All the healthcare providers involved in intra-partum and immediate post-partum care, irrespective of their qualifications. They were offered to participate in the study and we excluded healthcare providers who were in the health facility for their qualifying internship.

For peer review only - http://bmjopen.bmj.com/site/about/guidelines.xhtml

- All the pregnant women in labour, admitted without c-section or referral needed to be identified at admission. We consecutively recruited all eligible pregnant women during the data collection period in each health facility. Exclusion criteria were being aged <18 years in both countries, pregnant women admitted at an advanced stage of the labour (third stage) with frequent (≤5-min apart) and intense uterine contractions that would prevent them from providing valid informed consent.

The sample size of pregnant women was calculated using the conservative proportion of 50% adherence to each essential best practice (EBP) with a precision of 5% and type I error of 5%. To account for the clustering nature of the data, the calculated sample size was adjusted using a design effect based on an intra-class correlation coefficient of 0.01[21] and a size of 15 observations per group. The minimum sample size was estimated to be 425 pregnant women to be included in the observation per pause-point in each country, giving a minimum total number of 1700 of observations per country.

Data collection tools and processes

Data collectors with a medical background (medical students, midwives) and who had undergone a two-day training including in study-room role plays and a field testing of the study tools, were appointed in the delivery room to directly observe and document healthcare providers' adherence to EBPs at four critical points: admission, before the woman starts pushing, immediately post-partum (soon after birth up to ≤ 1h), and before discharge from the health facility. During the field test, a pair of data collectors had to observe at least two pause-points of the same birth event along with a supervisor. Data collectors' measurements were compared within each pair of data collectors and to the measurements of the supervisor. The observed variations were discussed.

For peer review only - http://bmjopen.bmj.com/site/about/guidelines.xhtml

The EBPs included in this study were derived from the World Health Organization Safe Childbirth Checklist (WHO-SCC)[22] complemented by a list of other validated quality indicators for SSA[16] (see Table 1). A total of 34 EBPs were observed during each birth event. Adherence to each EBP was measured using a binary yes/no variable. The observation was strictly passive with minimum interaction between observer and healthcare workers and patients. The data collectors were only allowed to intervene in the event of a life-threatening complication. The data collection took place from 12 November to 20 December 2018 and lasted for up to five days in each health facility. Observations were conducted every day of the week and at any time without discontinuation, provided an eligible participant was present. A data collector could observe all or part of the four pause-points for each birth event. However, data pertaining to the same critical juncture were fully collected by the same data collector. Thus, once a data collector had initiated the observation for a given pause-point, he/she could get off work only after having filled in all the items pertaining to that pause point.

For peer review only - http://bmjopen.bmj.com/site/about/guidelines.xhtml

Table 1: Essential best practices (EBPs) to be observed for health workers' compliance at each pause-point

N°	Admission	Before pushing	Soon after birth	Before discharge
1	○ Washes his/her hands before examination	○ Sterile gloves available	○ Clamps or ties cord correctly	○ Assesses the mother for vaginal bleeding
2	○ Wears sterile gloves before vaginal examination	○ Soap and clean water available	○ Immediately dries the baby with clean towel	○ Assesses the baby for the quality of breastfeeding
3	○ Asks for vaginal bleedings	○ Prepares a prefilled syringe with uterotonics for AMTSL	○ Assesses for vaginal and perineal lacerations	○ Discusses postpartum family planning with the woman/couple
4	○ Asks for vision blurred	○ Clean towel available	○ Assesses for placenta completeness	○ Plans postpartum visits and makes sure they will consult in the event of a complication
5	○ Checks HIV status	○ Sterile blade or scissor available	○ Assesses new born for need of reference	
6	○ Measures blood pressure	○ Face mask available	○ Takes mother vital signs within 15 minutes after	
7	○ Measures pulse	○ Suction device available	○ Palpates uterus within 15 minutes after birth	
8	○ Uses partograph where indicated	○ Assistant birth attendant identified and ready to intervene-	○ Initiates breastfeeding within 1 hour	
9	○ Measures temperature-yes		○ Skin-to-skin contact initiated within 1 hour	
10	○ Assesses for need of referral		○ Informs companion/woman to call if complications	
11	○ Encourages companion to assist			
12	○ Informs companion/woman to call in case of complications			

For peer review only - http://bmjopen.bmj.com/site/about/guidelines.xhtml

Data analysis

Descriptive statistics were computed using means and standard deviations or medians and interquartile ranges for numerical variables, and proportions were used to describe categorical variables. We calculated the level of adherence to each EBP as the proportion of childbirth events in which the EBP was effectively performed. These proportions were computed along with their 95% confidence intervals (CIs). For each pause-point, based on complete cases, a quality score was computed as the number of EBPs effectively performed at each birth event. We further used the Wilcoxon Ranksum test to compare quality scores at the four clinical junctures between PHCFs and hospitals in each country. Because the study finally included varied numbers of observations per health facility, all the analyses were weighted for the clinic size. A p-value <0.05 indicated statistical significance.

Ethics

The research protocol obtained ethical approvals from the national ethics review committees of Burkina Faso (reference number 2017-4-043) and Côte d'Ivoire (161-18/MSHP/CNE SVS-Kp). It was also approved by the ethics review committee of the WHO (protocol ID ERC.0002951). Written informed consent was obtained from all participants of the study. Data were collected, managed, and analysed in a way to ensure the confidentiality of study participants.

Patient and public involvement

There has been no patient and/or public involvement in the study design, data collection, data analysis, and writing of this research.

For peer review only

For peer review only - http://bmjopen.bmj.com/site/about/guidelines.xhtml

RESULTS

A total of 73 and 69 health facilities in Burkina Faso and Côte d'Ivoire, respectively, were included in the study. In total, 532 and 627 pregnant women in labour gave informed consent to be included in the observations for pause-point I in Burkina Faso and Côte d'Ivoire, respectively. Once included in the observation (pause-point I), a woman and thereafter her newborn were observed through the subsequent pause-points unless they were referred to another health facility or transferred to a different service. Another reason why a mother–newborn pair could not be observed at the fourth pause-point was them still being in the health facility at the time the data collectors had completed their overall stay in that particular facility. The number of birth events observed by pause-point by country and by health facility type are presented in Table 2. As stated above, the reduction in participant numbers from each pause-point to the next was a result of referral, transfer, and not yet being discharged from the health facility at the end of observation period.

Compliance with EBPs at admission

In Burkina Faso, adherence to EBPs ranged from 7.96% (95% CI: 5.66–11.06; wears sterile gloves for vaginal examination) to 99.27% (95% CI: 97.74–99.76; assesses the woman for need of referral). The adherence was significantly higher in referral hospitals as compared to PHCFs for the following EBPs: asks about vaginal bleeding ($p<0.05$), asks about blurred vision ($p<0.01$), measures blood pressure ($p<0.001$), measures pulse ($p<0.01$), and measures temperature ($p<0.05$). Adherence was significantly higher in PHCFs for the EBP 'encourages companion/woman to call in case of complication' ($p<0.01$).

In Côte d'Ivoire, adherence to EBPs ranged from 6.71% (95% CI: 3.94–11.20; washes his/her hands before examination) to 84.97% (95% CI: 81.24–88.09; assesses the woman for need of referral). The compliance was significantly higher in PHCFs than referral hospitals for the

For peer review only - http://bmjopen.bmj.com/site/about/guidelines.xhtml

following EBPs: asks about vaginal bleeding ($p<0.01$), measures blood pressure ($p<0.001$), measures pulse ($p<0.01$), measures temperature ($p<0.01$), and encourages companion/woman to call in case of complication ($p<0.001$).

Compliance with EBPs at the immediate pre-pushing phase

In Burkina Faso, all EBPs were implemented in more than half of the deliveries that took place in referral hospitals. The same was observed in PHCFs, except for availability of bag and mask, which was observed in 39.34% (95% CI: 34.38–44.53) of the deliveries. The adherence was significantly higher in referral hospitals than PHCFs for three EBPs: prepares pre-filled syringe with uterotonics for active management of the third stage of labour (AMTSL) ($p<0.05$), bag and mask available ($p<0.001$), and assistant birth attendant ready to intervene if needed ($p<0.001$).

In Côte d'Ivoire, except for availability of bag and mask and availability of suction device, all other EBPs were adhered to in more than half of the deliveries in both types of health facilities. Bag and mask were available in 5.67% (95% CI: 3.68–8.77) and 18.74% (95% CI: 12.17–27.69) of the deliveries in PHCFs and referral hospitals, respectively. Availability of suction device ($p<0.001$) and sterile blade or scissor ($p<0.01$) were also better in referral hospitals than PHCFs and preparation of a pre-filled syringe with uterotonics for AMTSL was best adhered to in PHCFs ($p<0.001$).

Compliance to EBPs immediately post-partum

In Burkina Faso, adherence was above 50% for all EBPs ranging from 51.86% (95% CI: 46.58–57.10; takes vital signs within 15 min) to 99.71% (95% CI: 98.67 to EBPs 99.94; assesses the newborn for need of referral). Significantly higher adherence was observed in referral hospitals regarding encouraging companion/woman to call in case of complication ($p<0.01$).

For peer review only - http://bmjopen.bmj.com/site/about/guidelines.xhtml

In Côte d'Ivoire, adherence ranged from 19.66% (95% CI: 15.49–24.61; clamps or ties cord correctly) to 98.5% (95% CI: 96.91–99.28; assesses for vaginal or perineal lacerations). The adherence was significantly higher in PHCFs for the following EBPs: Assesses for vaginal and perineal lacerations ($p<0.01$), assesses newborn for need of referral ($p<0.01$), palpates uterus within 15 min after birth ($p<0.05$), initiates breastfeeding within 1 h ($p<0.01$), and skin-to-skin contact initiated within 1 h ($p<0.001$).

Compliance to EBPs before discharge from health facility

When leaving the facility, 47.14% (95% CI: 37.09–47.36) of women in Burkina Faso were offered a discussion on post-partum family planning with a significantly higher performance in referral hospitals ($p<0.001$). In Cote d'Ivoire, the quality of breastfeeding was assessed in 26.48% (95% CI: 21.46–32.19) cases and was best adhered to in PHCFs ($p<0.001$).

The levels of adherence to all EBPs per pause-point, country, and health facility type are presented in Table 2.

For peer review only - http://bmjopen.bmj.com/site/about/guidelines.xhtml

Table 2: Compliance to essential best practices by pause-point and by health facility type in Burkina Faso and Côte d'Ivoire.

Essential best practices	Burkina Faso				Côte d'Ivoire			
	PHCF		RH		PHCF		RH	
	n/N	% (95% CI)	n/N	% (95% CI)	n /N	% (95% CI)	n/N	% (95% CI)
**Admission**	**428**		**104**		**513**		**114**	
Washes his/her hands before examination	86/420	20.50 (16.72-24.86)	20/101	19.02(12.48-27.89)	29/501	06.72(03.94-11.24)	5/114	05.00(02.10-11.45)
Wears sterile gloves before vaginal examination	33/425	07.94(05.64-11.07)	11/103	10.73(06.03-18.39)	49/500	07.03(05.23-09.39)	6/114	05.60(02.52-11.96)
Asks for vaginal bleedings	181/422	**41.62(36.57-46.84)**	55/104	**54.18(44.46-63.58)**	144/504	**33.97(28.96-39.38)**	23/114	**20.40(13.90-28.92)**
Asks for vision blurred	160/422	**37.04(32.12-42.24)**	53/104	**52.51(42.82-62.01)**	122/504	27.67(23.29-32.52)	28/114	25.40(18.12-34.38)
Checks HIV status	333/425	79.25(75.00-82.94)	89/104	86.62(78.85-91.83)	344/497	66.08(60.69-71.08)	73/113	65.24(55.94-73.51)
Measures blood pressure	305/428	**72.32(67.48-76.68)**	94/103	**90.95(83.45-95.25)**	414/509	**81.21(76.66-85.04)**	70/114	**63.63(54.42-71.94)**
Measures pulse	170/428	**39.87(34.85-45.10)**	54/102	**54.36(44.55-63.85)**	230/506	**48.57(43.18-54.00)**	35/114	**33.56(25.25-43.02)**
Uses partograph where indicated	225/428	53.11(47.90-58.25)	53/104	50.36(40.73-59.97)	90/508	17.72(14.04-22.13)	24/114	23.80(16.57-32.94)
Measures temperature	277/428	**65.64(60.50-70.43)**	79/104	**76.61(67.49-83.79)**	207/513	**42.90(37.60-48.36)**	29/114	**27.47(18.83-36.70)**
Assesses for need of referral	425/428	99.26(97.74-99.76)	104/104	100	441/512	**85.04(81.27-88.16)**	89/114	**76.6(67.65-83.67)**
Encourages companion to assist to the childbirth	176/422	**41.47(36.49-46.63)**	26/103	**24.59(17.22-33.82)**	43/474	08.64(06.22-11.88)	11/109	11.14(06.27-19.03)
Companion/woman informed to call if complication	326/424	86.15(82.06-89.42)	101/104	96.7(90.07-98.96)	277/472	**66.07(60.93-70.86)**	45/105	**47.56(38.03-57.26)**
**Before pushing or before c-section**	**416**		**99**		**477**		**109**	
Sterile gloves available	405/416	97.65(95.78-98.71)	96/99	96.70(90.13-98.94)	458/477	93.21(87.48-96.43)	104/109	95.54(89.61-98.16)
Soap and clean water available	259/416	63.42(58.23-68.31)	68/99	70.37(60.73-78.49)	365/477	71.15(65.60-76.12)	83/109	75.65(66.56-82.91)
Prepares a prefilled syringe with uterotonics for AMTSL	348/416	**83.19(79.05-86.65)**	92/99	**92.85(85.71-96.56)**	372/477	**83.74(80.22-86.74)**	69/109	**68.05(58.93-75.96)**
Clean towel available	389/416	94.41(91.93-96.17)	96/99	96.90(90.80-99.00)	416/477	85.97(82.23-89.02)	102/109	92.92(85.87-96.60)
Sterile blade or scissor available	400/416	96.56(94.42-97.90)	98/99	99.00(93.21-99.86)	374/477	**76.15(70.79-80.79)**	98/109	**88.55(80.54-93.53)**
Bag and mask available	168/416	**39.15(34.16-44.37)**	76/99	**76.12(66.58-83.60)**	22/477	**05.67(03.63-08.77)**	18/109	**18.73(12.17-27.69)**
Suction device available	312/416	74.57(69.54-79.02)	80/99	80.17(70.96-86.99)	99/477	**17.89(14.57-21.76)**	60/109	**58.06(48.55-67.00)**
Assistant birth attendant ready to intervene	206/416	**49.97(44.69-55.24)**	76/99	**76.13(66.58-83.62)**	415/476	84.87(80.55-88.37)	95/109	88.14(80.86-92.89)
**Soon after birth (within one hour)**	**415**		**99**		**477**		**109**	

For peer review only - http://bmjopen.bmj.com/site/about/guidelines.xhtml

	Burkina Faso				Côte d'Ivoire			
Clamps or ties cord correctly	260/415	61.72(56.40-66.77)	56/99	58.42(48.45-67.74)	82/477	**19.57(15.33-24.57)**	32/109	**32.46(24.08-42.14)**
Immediately dries the baby with clean towel	412/415	99.23(97.60-99.75)	99/99	100	441/477	92.37(89.52-94.49)	103/109	93.96(87.18-97.27)
Assesses for vaginal and perineal lacerations	393/415	94.81(92.11-96.63)	95/99	96.18(90.23-98.57)	470/477	**99.54(96.91-99.31)**	102/109	**93.13(86.24-96.70)**
Assesses for placenta completeness	235/414	55.17(49.83-60.40)	45/99	47.42(37.65-57.40)	171/476	37.02(32.05-42.28)	33/109	33.18(24.74-42.86)
Assesses new-born for need of reference	413/415	99.71(98.65-99.94)	98/99	99.00(93.21-99.86)	418/477	**86.24(82.47-89.30)**	82/108	**74.46(65.12-81.99)**
Takes mother vital signs within 15 minutes after	208/415	51.82(46.52-57.09)	60/99	58.38(48.26-67.85)	234/477	47.01(41.69-52.40)	38/108	38.44(29.52-48.21)
Palpates uterus within 15 minutes after birth	337/415	83.78(79.75-87.14)	79/99	79.44(70.16-86.39)	289/477	**59.11(53.44-64.55)**	45/108	**44.80(35.61-54.53)**
Initiates breastfeeding within 1 hour	298/395	75.85(71.01-80.06)	63/93	67.36(57.08-76.20)	184/434	**48.24(42.46-54.06)**	28/100	**30.14(21.72-40.16)**
Skin-to-skin contact initiated within 1 hour	331/406	81.81(76.98-85-81)	74/97	76.02(66.43-83.56)	275/465	**62.14(56.94-67.09)**	34/103	**35.09(26.29-45.03)**
Informs companion/woman to call if complications	350/415	**85.15(80.92-88.57)**	95/99	**95.80(89.20-98.44)**	365/474	78.03(73.47-82.00)	74/105	70.86(61.39-78.81)
**Before discharge from the health facility**	**411**		**93**		**474**		**106**	
Assesses the mother for vaginal bleeding	291/408	70.82(65.54-75.59)	73/93	79.82(70.59-86.69)	310/474	68.68(63.46-73.46)	64/105	64.62(55.10-73.10)
Assesses the baby for the quality of breastfeeding	227/408	55.56(50.16-60.84)	49/92	52.46(42.14-62.59)	129/474	**26.60(21.55-32.35)**	6/106	**06.43(02.92-13.58)**
Discusses postpartum family planning with the woman/couple	173/409	**42.01(36.94-47.26)**	64/92	**67.24(56.67-76.31)**	169/473	29.10(24.52-34.12)	35/106	30.19(22.27-39.50)
Plans postpartum visits and informs to consult if complications	349/409	**84.28(79.20-88.30)**	86/92	**93.21(85.60-96.95)**	380/474	**77.68(72.94-81.81)**	73/106	**67.85(58.20-76.18)**

PHCF: Primary Health Care Facility; RH: Referral Hospital

For peer review only

For peer review only - http://bmjopen.bmj.com/site/about/guidelines.xhtml

The overall mean quality scores were consistently higher in referral hospitals than PHCFs in Burkina Faso at all pause-points (p<0.01) except immediately post-partum when there was no significant difference. In Côte d'Ivoire, the overall quality score was better in PHCFs at admission (p<0.001), immediately post-partum (p<0.001), and before discharge from the health facility (p<0.01). It was higher in referral hospitals at the immediate pre-pushing phase (p<0.01). The distribution of the quality scores by pause-point and by country is depicted in Figure 1.

DISCUSSION

In this study, we assessed healthcare providers' adherence to EBPs in delivery care using direct observation. Our results showed a wide diversity in adherence to EBPs ranging from very low to very high. Overall, adherence to EBPs tended to be better in referral hospitals as compared to PHCFs in Burkina Faso. In Côte d'Ivoire, the compliance was higher in PHCFs than referral hospitals. Important quality gaps were shown in both countries. Hand hygiene (washing hands before examination and wearing sterile gloves for each vaginal examination) practices were particularly very poor in both countries despite the widespread availability of hand hygiene facilities in both countries (hand washing facilities with soap and clean water were found in 94.74% and 74.96% of the maternity wards of surveyed healthcare facilities in Burkina Faso and Côte d'Ivoire, respectively). With the equipment available, adherence to hygiene routines is a fundamental and non-demanding practice for prevention of infections.[23,24] Yet, non-compliance is widespread and remains a source of concern globally.[25,26]

History-taking of critical information such as vaginal bleeding or blurred vision during pregnancy was sub-optimal in Burkina Faso and poor in Côte d'Ivoire. Omission of blood pressure measurement was common, and occurred in one out of four women in Côte d'Ivoire.

For peer review only

For peer review only - http://bmjopen.bmj.com/site/about/guidelines.xhtml

Temperature was not measured in two-thirds of the women in labour, missing an opportunity to identify ongoing episodes of infection.

These gaps in initial assessment can cause at-risk patients or prevailing complications to go undetected. It is also noteworthy that, despite being an ancient and long-systematized intervention, a partograph to monitor labour was used during less than half of the deliveries in both countries. The low utilization of the partograph and its poor quality when used are well recognized and have been widely reported in the literature.[27–29]

Assessment of vaginal bleeding and perineal lacerations and preparation of a prefilled syringe with uterotonics for AMTSL were satisfactorily adhered to in both countries (>75%). These are important practices for the prevention, early detection, and management of post-partum haemorrhage (PPH), a leading cause of maternal death.[4] Bedside availability of prepared oxytocin improves its timely administration.[30] Increased compliance to these standards may be the result of rising awareness of the importance of PPH as a leading cause of death and the emphasis put on its prevention and management in both initial and refresher trainings for healthcare providers.

Implementation of other practices equally relevant to PPH: the performance of 'assessment of the completeness of the placenta' and 'monitoring of maternal vital signs' was however very low, and only complied with for around half of the women in Burkina Faso for both EBPs, and to one-third and three-sevenths of the women, respectively, in Côte d'Ivoire. These are life-saving low-cost practices, but require health-worker awareness and knowledge on the purpose and rationale for providing these services, in addition to clinical skills in executing them. The low proportion of women in both countries that received these interventions represent unmet needs of urgent action to address these cost-effective practices. Our findings also highlight the complexity of the peri- and immediate post-partum care, wherein a series of

For peer review only

For peer review only - http://bmjopen.bmj.com/site/about/guidelines.xhtml

equally important steps need to be taken to minimize risks of maternal morbidity, and how failure to address all of these puts women at risk of severe consequences.

Thermal management of newborns was satisfactorily adhered to in both countries through the availability of clean towels before the woman started pushing and drying the newborn immediately post-partum. Compliance with other lifesaving interventions for newborns (skin-to-skin contact, early initiation of breastfeeding) was beyond 75% women in Burkina Faso and barely half of the women in Côte d'Ivoire.

Overall, supportive services (encouraging companion to assist in the delivery, identifying and preparing an assistant birth attendant ready to help) at any point in time tend to be less adhered to as compared to other standards of care.

The health facilities in both countries rarely used the opportunity to provide post-partum contraceptive advice to the patients. The same has been previously reported[21] and is suggestive of health workers' propensity to give precedence to interventions needed to prevent or treat imminent and tangible complications. Given the large unmet needs of modern contraceptives in both countries, implementation of post-partum family planning programmes is an important strategy to close the gap. Knowledge translation and follow-up of patients is needed between healthcare workers engaged in intra-partum care and family planning.

Noticeably, a significant proportion of women and or companions received information related to potential complications and signs of danger and what to do in case they occur in both countries from admission to discharge. Apart from the immediate post-partum period wherein there was no significant difference, compliance with standards in Burkina Faso tended to be better at all junctures in referral hospitals than PHCFs. Such a systematic difference was not observed in Côte d'Ivoire. This difference may be because in Burkina Faso, there is a wider existence of categories of auxiliary nurses and auxiliary midwives who

For peer review only

For peer review only - http://bmjopen.bmj.com/site/about/guidelines.xhtml

predominantly work in PHCFs, while in Côte d'Ivoire, deliveries are attended to by nurses and midwives at PHCFs as well as referral hospitals.

Study limitations

In interpreting our results, some limitations should be considered. We carried out a direct observation of facility-based deliveries in two selected regions and the findings may not be generalizable to each individual country. Although we did not provide information to healthcare providers on the items being observed, we cannot rule out the Hawthorne effect and some may have changed their healthcare practices during the study period. The data collection was carried out by eight data collectors in each country. Although they were trained by the same research team to standardize processes and judgements, we cannot rule out issues related to inter-rater reliability. Finally, the non-compliance to a given standard does not always imply that the observed healthcare provider failed to do so. In some cases, the supplies needed to administer the intervention may have been unavailable.

CONCLUSION

Overall, compliance to EBPs during delivery was insufficient and important gaps in quality of care were identified. A significant number of women in Burkina Faso and Cote d'Ivoire missed out on important interventions, especially those related to infection prevention and control and prevention of PPH. In addition, there were lost opportunities in relation to implementation of post-partum contraceptive strategies. Conditional on the availability of supplies, the investigated EBPs were relatively low cost and easy to implement. Quality-improvement interventions in the form of reminder tools for healthcare providers, such as the WHO-SSC have the potential to sustain currently performed best practices while filling the gaps that persist.

For peer review only

Authors' contributions

All authors meet the criteria recommended by the International Committee of Medical Journal Editors (ICMJE). All authors made substantial contributions to conception and design, acquisition of data or analysis and interpretation of data. SK,MT,MLA conceived the study design. MT analysed the data and proposed the first draft of the paper. SK, MLA, RKK, MWY, AT, FBS, ASC and AT revised and contributed to the successive drafts. The final version of the paper was revised and approved by all the authors.

Acknowledgment

We are grateful to WHO Human Reproduction Program (WHO/HRP) for funding the research.

Data availability statement

The data presented in this study are from the Checklist study conducted in Burkina Faso and Côte d'Ivoire. Data can be accessed upon request sent to the principal investigator of the study, Prof Seni Kouanda at senikouanda@gmail.com. Access will be granted only after careful and due consideration of the compliance with the ethics requirements and the data policy of the Institut de Recherche en Sciences de la Santé.

Funding

This work was supported by WHO/HRP. The funders have no role in the design of the research, nor they have in the collection, analysis, and interpretation of the data.

Competing interests

All authors declare that they have no competing interests.

For peer review only

For peer review only - http://bmjopen.bmj.com/site/about/guidelines.xhtml

REFERENCES

1. Alkema L, Chou D, Hogan D, Zhang S, Moller A-B, Gemmill A, Fat DM, Boerma T, Temmerman M, Mathers C, Say L. Global, regional, and national levels and trends in maternal mortality between 1990 and 2015, with scenario-based projections to 2030: a systematic analysis by the UN Maternal Mortality Estimation Inter-Agency Group. The Lancet. 2016 Jan;387(10017):462–74.
2. Victora CG, Requejo JH, Barros AJD, Berman P, Bhutta Z, Boerma T, Chopra M, de Francisco A, Daelmans B, Hazel E, Lawn J, Maliqi B, Newby H, Bryce J. Countdown to 2015: a decade of tracking progress for maternal, newborn, and child survival. The Lancet. 2016 May;387(10032):2049–59.
3. World Health Organization, UNICEF, United Nations, Department of Economic and Social Affairs, Population Division, World Bank. Trends in maternal mortality: 1990 to 2015 : estimates by WHO, UNICEF, UNFPA, World Bank Group and the United Nations Population Division [Internet]. 2015 [cited 2017 Dec 17]. Available from: http://www.who.int/reproductivehealth/publications/monitoring/maternal-mortality-2015/en/
4. Ronsmans C, Graham WJ, Lancet Maternal Survival Series steering group. Maternal mortality: who, when, where, and why. Lancet. 2006 Sep 30;368(9542):1189–200.
5. Bullough C, Meda N, Makowiecka K, Ronsmans C, Achadi EL, Hussein J. REVIEW: Current strategies for the reduction of maternal mortality: REVIEW. BJOG Int J Obstet Gynaecol. 2005 Jun 29;112(9):1180–8.
6. World Health Organization. Making pregnancy safer the critical role of the skilled attendant: a joint statement by WHO, ICM and FIGO. Geneva: World health organization; 2004.
7. Udo IE, Doctor HV. Trends in Health Facility Births in sub-Saharan Africa: An Analysis of Lessons Learned under the Millennium Development Goal Framework. Afr J Reprod Health [Internet]. 2017 May 22 [cited 2017 Dec 18];20(3). Available from: https://www.ajrh.info/index.php/ajrh/article/view/175
8. Doctor HV, Nkhana-Salimu S, Abdulsalam-Anibilowo M. Health facility delivery in sub-Saharan Africa: successes, challenges, and implications for the 2030 development agenda. BMC Public Health. 2018 Dec;18(1):765.
9. Ekirapa-Kiracho E, Waiswa P, Rahman MH, Makumbi F, Kiwanuka N, Okui O, Rutebemberwa E, Bua J, Mutebi A, Nalwadda G, Serwadda D, Pariyo GW, Peters DH. Increasing access to institutional deliveries using demand and supply side incentives: early results from a quasi-experimental study. BMC Int Health Hum Rights. 2011;11 Suppl 1:S11.
10. Kongnyuy EJ, Mlava G, van den Broek N. Facility-Based Maternal Death Review In Three Districts In The Central Region of Malawi. Womens Health Issues. 2009 Jan;19(1):14–20.
11. van den Broek N, Graham W. Quality of care for maternal and newborn health: the neglected agenda: Quality of care for maternal and newborn health. BJOG Int J Obstet Gynaecol. 2009 Oct;116:18–21.
12. Chan M, Director-General W. QUALITY OF CARE IN THE CONTEXT OF UNIVERSAL HEALTH COVERAGE (UHC). :2.
13. Admasu K, Haile-Mariam A, Bailey P. Indicators for availability, utilization, and quality of emergency obstetric care in Ethiopia, 2008. Int J Gynecol Obstet. 2011 Oct;115(1):101–5.
14. Wilunda C, Oyerinde K, Putoto G, Lochoro P, Dall'Oglio G, Manenti F, Segafredo G, Atzori A, Criel B, Panza A, Quaglio G. Availability, utilisation and quality of maternal and neonatal health care services in Karamoja region, Uganda: a health facility-based survey. Reprod Health [Internet]. 2015 Dec [cited 2017 Dec 18];12(1). Available from:

For peer review only - http://bmjopen.bmj.com/site/about/guidelines.xhtml

http://reproductive-health-journal.biomedcentral.com/articles/10.1186/s12978-015-0018-7

15. Baguiya A, Meda IB, Millogo T, Kourouma M, Mouniri H, Kouanda S. Availability and utilization of obstetric and newborn care in Guinea: A national needs assessment. Int J Gynaecol Obstet Off Organ Int Fed Gynaecol Obstet. 2016 Nov;135 Suppl 1:S2–6.

16. Tripathi V, Stanton C, Strobino D, Bartlett L. Development and Validation of an Index to Measure the Quality of Facility-Based Labor and Delivery Care Processes in Sub-Saharan Africa. Duan T, editor. PLOS ONE. 2015 Jun 24;10(6):e0129491.

17. Institut National de la Statistique et de la Démographie (INSD), ICF International. Enquête Démographique et de Santé et à Indicateurs Multiples du Burkina Faso 2010. Calverton, Maryland, USA; 2012.

18. Institut National de la Statistique (INS), ICF International. Enquête Démographique et de Santé et à Indicateurs Multiples de Côte d'Ivoire 2011-2012. Calverton, Maryland, USA : INSD et ORC Macro.; 2012.

19. Ministère de la Santé. Annuaire Statistique 2015 [Internet]. Ouagadougou; 2016 Mar. Available from: http://www.sante.gov.bf/index.php?option=com_edocman&task=document.viewdoc&id=113&Itemid=1123

20. Alkema L, Chou D, Hogan D, Zhang S, Moller A-B, Gemmill A, Fat DM, Boerma T, Temmerman M, Mathers C, Say L. Global, regional, and national levels and trends in maternal mortality between 1990 and 2015, with scenario-based projections to 2030: a systematic analysis by the UN Maternal Mortality Estimation Inter-Agency Group. The Lancet. 2016 Jan;387(10017):462–74.

21. Kumar S, Yadav V, Balasubramaniam S, Jain Y, Joshi CS, Saran K, Sood B. Effectiveness of the WHO SCC on improving adherence to essential practices during childbirth, in resource constrained settings. BMC Pregnancy Childbirth [Internet]. 2016 Dec [cited 2017 Jan 17];16(1). Available from: http://bmcpregnancychildbirth.biomedcentral.com/articles/10.1186/s12884-016-1139-x

22. Spector JM, Lashoher A, Agrawal P, Lemer C, Dziekan G, Bahl R, Mathai M, Merialdi M, Berry W, Gawande AA. Designing the WHO Safe Childbirth Checklist program to improve quality of care at childbirth. Int J Gynecol Obstet. 2013 Aug;122(2):164–8.

23. Allegranzi B, Pittet D. Role of hand hygiene in healthcare-associated infection prevention. J Hosp Infect. 2009 Dec;73(4):305–15.

24. Pittet D, Allegranzi B, Storr J. The WHO Clean Care is Safer Care programme: Field-testing to enhance sustainability and spread of hand hygiene improvements. J Infect Public Health. 2008;1(1):4–10.

25. Musuuza JS, Barker A, Ngam C, Vellardita L, Safdar N. Assessment of Fidelity in Interventions to Improve Hand Hygiene of Healthcare Workers: A Systematic Review. Infect Control Hosp Epidemiol. 2016 May;37(5):567–75.

26. Anargh V, Singh H, Kulkarni A, Kotwal A, Mahen A. Hand hygiene practices among health care workers (HCWs) in a tertiary care facility in Pune. Med J Armed Forces India. 2013 Jan;69(1):54–6.

27. Asibong U, Okokon IB, Agan TU, Oku A, Opiah M, Essien EJ, Monjok E. The use of the partograph in labor monitoring: a cross-sectional study among obstetric caregivers in General Hospital, Calabar, Cross River State, Nigeria. Int J Womens Health. 2014;6:873–80.

28. Opiah MM, Ofi AB, Essien EJ, Monjok E. Knowledge and Utilization of the Partograph among Midwives in the Niger Delta Region of Nigeria. Afr J Reprod Health Rev Afr Santé Reprod. 2012;16(1):125–32.

29. Mandiwa C, Zamawe C. Documentation of the partograph in assessing the progress of labour by health care providers in Malawi's South-West zone. Reprod Health. 2017 Dec;14(1):134.

For peer review only

For peer review only - http://bmjopen.bmj.com/site/about/guidelines.xhtml

30. Moucheraud C, Gass J, Lipsitz S, Spector J, Agrawal P, Hirschhorn LR, Gawande A, Kodkany B. Bedside Availability of Prepared Oxytocin and Rapid Administration After Delivery to Prevent Postpartum Hemorrhage: An Observational Study in Karnataka, India. Glob Health Sci Pract. 2015 Jun;3(2):300–4.

For peer review only

For peer review only - http://bmjopen.bmj.com/site/about/guidelines.xhtml

Figure legends

Figure 1: The boxplots depict the overall scores of quality of care computed for each pause-point by health facility type and by country. The maximum quality score for any pause-point is equal to the number of essential best practices observed at that pause-point.

For peer review only

For peer review only - http://bmjopen.bmj.com/site/about/guidelines.xhtml

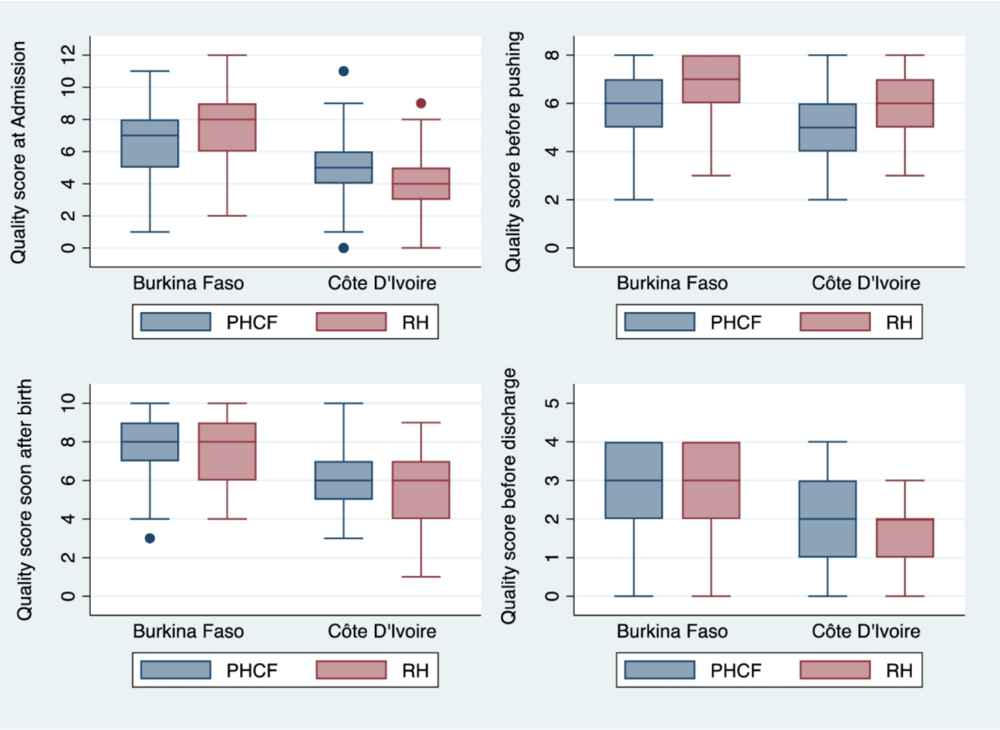

Figure 1: Distribution of quality scores per country and pause-points.

Figure legends

Figure 1: The boxplots depict the overall scores of quality of care computed for each pause-point by health facility type and by country. The maximum quality score for any pause-point is equal to the number of essential best practices observed at that pause-point.

The boxplots depict the overall scores of quality of care computed for each pause-point by health facility type and by country. The maximum quality score for any pause-point is equal to the number of essential best practices observed at that pause-point.

165x168mm (300 x 300 DPI)

STROBE Statement—Checklist of items that should be included in reports of ***cross-sectional studies***

	Item No	Recommendation	Page No
**Title and abstract**	1	(*a*) Indicate the study's design with a commonly used term in the title or the abstract	1 &2
		(*b*) Provide in the abstract an informative and balanced summary of what was done and what was found	2
**Introduction**			
Background/rationale	2	Explain the scientific background and rationale for the investigation being reported	4
Objectives	3	State specific objectives, including any prespecified hypotheses	4
**Methods**			
Study design	4	Present key elements of study design early in the paper	5
Setting	5	Describe the setting, locations, and relevant dates, including periods of recruitment, exposure, follow-up, and data collection	5, 7
Participants	6	(*a*) Give the eligibility criteria, and the sources and methods of selection of participants	6
Variables	7	Clearly define all outcomes, exposures, predictors, potential confounders, and effect modifiers. Give diagnostic criteria, if applicable	8
Data sources/ measurement	8*	For each variable of interest, give sources of data and details of methods of assessment (measurement). Describe comparability of assessment methods if there is more than one group	*7*
Bias	9	Describe any efforts to address potential sources of bias	7
Study size	10	Explain how the study size was arrived at	6 &7
Quantitative variables	11	Explain how quantitative variables were handled in the analyses. If applicable, describe which groupings were chosen and why	9
Statistical methods	12	(*a*) Describe all statistical methods, including those used to control for confounding	9
		(*b*) Describe any methods used to examine subgroups and interactions	NA
		(*c*) Explain how missing data were addressed	9
		(*d*) If applicable, describe analytical methods taking account of sampling strategy	
		(*e*) Describe any sensitivity analyses	
**Results**			
Participants	13*	(a) Report numbers of individuals at each stage of study—eg numbers potentially eligible, examined for eligibility, confirmed eligible, included in the study, completing follow-up, and analysed	9&10
		(b) Give reasons for non-participation at each stage	10
		(c) Consider use of a flow diagram	
Descriptive data	14*	(a) Give characteristics of study participants (eg demographic, clinical, social) and information on exposures and potential confounders	10
		(b) Indicate number of participants with missing data for each variable of interest	
Outcome data	15*	Report numbers of outcome events or summary measures	10 & 11

For peer review only

Main results	16	(*a*) Give unadjusted estimates and, if applicable, confounder-adjusted estimates and their precision (eg, 95% confidence interval). Make clear which confounders were adjusted for and why they were included	12
		(*b*) Report category boundaries when continuous variables were categorized	
		(*c*) If relevant, consider translating estimates of relative risk into absolute risk for a meaningful time period	
Other analyses	17	Report other analyses done—eg analyses of subgroups and interactions, and sensitivity analyses	
**Discussion**			
Key results	18	Summarise key results with reference to study objectives	12
Limitations	19	Discuss limitations of the study, taking into account sources of potential bias or imprecision. Discuss both direction and magnitude of any potential bias	14
Interpretation	20	Give a cautious overall interpretation of results considering objectives, limitations, multiplicity of analyses, results from similar studies, and other relevant evidence	12 & 13
Generalisability	21	Discuss the generalisability (external validity) of the study results	14
**Other information**			
Funding	22	Give the source of funding and the role of the funders for the present study and, if applicable, for the original study on which the present article is based	15

*Give information separately for exposed and unexposed groups.

Note: An Explanation and Elaboration article discusses each checklist item and gives methodological background and published examples of transparent reporting. The STROBE checklist is best used in conjunction with this article (freely available on the Web sites of PLoS Medicine at http://www.plosmedicine.org/, Annals of Internal Medicine at http://www.annals.org/, and Epidemiology at http://www.epidem.com/). Information on the STROBE Initiative is available at www.strobe-statement.org.

For peer review only - http://bmjopen.bmj.com/site/about/guidelines.xhtml

BMJ Open

Quality of maternal and newborn care in limited-resource settings: a facility-based cross-sectional study in Burkina Faso and Côte d’Ivoire

Journal:	*BMJ Open*
Manuscript ID	bmjopen-2019-036121.R2
Article Type:	Original research
Date Submitted by the Author:	05-May-2020
Complete List of Authors:	Millogo, Tieba; African Institute of Public Health, Epidemiology; Institut de Recherche en Sciences de la Sante<br>Agbre-Yace , Marie; Institut National de Santé Publique (INSP-Côte d’Ivoire)<br>Kourouma, Raissa; Institut National de Santé Publique (INSP-Côte d’Ivoire)<br>Yaméogo, W. Maurice; Institut de Recherche en Sciences de la Sante<br>Tano-Kamelan , Akoua ; Institut National de Santé Publique (INSP-Côte d’Ivoire)<br>Sissoko, Fatou; Institut de Recherche en Sciences de la Sante<br>Soltié-Coulibaly, Aminata; Institut National de Santé Publique (INSP-Côte d’Ivoire)<br>Thorson, Anna; Human Reproduction Program/World Health Organization (Geneva)<br>Kouanda, Seni; Institut de Recherche en Sciences de la Sante; African Institute of Public Health
**Primary Subject Heading**:	Public health
Secondary Subject Heading:	Health services research, Public health, Evidence based practice
Keywords:	Quality in health care < HEALTH SERVICES ADMINISTRATION & MANAGEMENT, PRIMARY CARE, PUBLIC HEALTH

SCHOLARONE™
Manuscripts

I, the Submitting Author has the right to grant and does grant on behalf of all authors of the Work (as defined in the below author licence), an exclusive licence and/or a non-exclusive licence for contributions from authors who are: i) UK Crown employees; ii) where BMJ has agreed a CC-BY licence shall apply, and/or iii) in accordance with the terms applicable for US Federal Government officers or employees acting as part of their official duties; on a worldwide, perpetual, irrevocable, royalty-free basis to BMJ Publishing Group Ltd ("BMJ") its licensees and where the relevant Journal is co-owned by BMJ to the co-owners of the Journal, to publish the Work in this journal and any other BMJ products and to exploit all rights, as set out in our licence.

The Submitting Author accepts and understands that any supply made under these terms is made by BMJ to the Submitting Author unless you are acting as an employee on behalf of your employer or a postgraduate student of an affiliated institution which is paying any applicable article publishing charge ("APC") for Open Access articles. Where the Submitting Author wishes to make the Work available on an Open Access basis (and intends to pay the relevant APC), the terms of reuse of such Open Access shall be governed by a Creative Commons licence – details of these licences and which Creative Commons licence will apply to this Work are set out in our licence referred to above.

Other than as permitted in any relevant BMJ Author's Self Archiving Policies, I confirm this Work has not been accepted for publication elsewhere, is not being considered for publication elsewhere and does not duplicate material already published. I confirm all authors consent to publication of this Work and authorise the granting of this licence.

For peer review only

Quality of maternal and newborn care in limited-resource settings: a facility-based cross-sectional study in Burkina Faso and Côte d'Ivoire

Millogo Tieba : African Institute of Public Health (IASP) & Institut de Recherche en Sciences de la Santé, Burkina Faso. millogorod@gmail.com ; tmillogo@iasp-bf.org .

Agbre-Yace Marie Laurette: Institut National de Santé Publique (INSP-Côte d'Ivoire). agbreyace@yahoo.fr .

Kourouma Raissa K : Institut National de Santé Publique (INSP-Côte d'Ivoire). kkouroum@gmail.com .

Yaméogo W. Maurice E : African Institute of Public Health (IASP) & Institut de Recherche en Sciences de la Santé, Burkina Faso. yamwamb@gmail.com .

Tano-Kamelan Akoua : Institut National de Santé Publique (INSP-Côte d'Ivoire). tanokamelan@yahoo.fr .

Sissoko Fatou B : Institut de Recherche en Science de la Santé (IRSS), Burkina Faso. bfatou.sissoko@yahoo.fr .

Soltié-Coulibaly Aminata: Institut National de Santé Publique (INSP-Côte d'Ivoire). soltieamy@yahoo.fr .

Thorson Anna : Human Reproduction Program/World Health Organization (Geneva). thorsona@who.int .

Kouanda Seni : Institut de Recherche en Sciences de la Santé, Burkina Faso & African Institute of Public Health (IASP). senikouanda@gmail.com .

Correspondence : **Millogo Tieba** : African Institute of Public Health. 12 BP 199 Ouagadougou. Burkina Faso. millogorod@gmail.com ; tmillogo@iasp-bf.org . Cel:+22671910013.

For peer review only

Abstract word count : 298
Manuscript word count: 3785

For peer review only - http://bmjopen.bmj.com/site/about/guidelines.xhtml

ABSTRACT

Objective: To assess and compare the quality of intra-partum and immediate post-partum care across levels of health care in Burkina Faso and Côte d'Ivoire using validated process indicators.

Design: Health-facility-based cross-sectional study with direct observation of healthcare workers' practices while caring for mother–newborn pairs during intra-partum and immediate post-partum periods.

Setting: Primary healthcare facilities and their corresponding referral hospitals in the Central-North region in Burkina Faso and the Agneby-Tiassa-Mé region in Côte d'Ivoire.

Participants: Healthcare providers who care for mother–newborn pairs during intra-partum and immediate post-partum periods, the labouring women, and their newborns after childbirth.

Main Outcome Measure(s): Adherence to essential best practices (EBPs) at four pause-points in each birth event and the overall quality score based on the level of adherence to the set of EBPs observed for a selected pause-point.

Results: A total of 532 and 627 laboring women were included in Burkina Faso and Côte d'Ivoire, respectively. Overall, the compliance with EBPs was insufficient at all the four pause-points, even though it varied widely from one EBP to another. The adherence was very low with respect to hand hygiene practices: the care provider wore sterile gloves for vaginal examination in only 7.96% cases (95% confidence interval (CI): 5.66–11.06) in Burkina Faso and the care provider washed hands before examination in 6.71% cases (95% CI: 3.94–11.20) in Côte d'Ivoire. The adherence was very high with respect to thermal management of newborns in both countries (>90%). The overall mean quality scores were consistently higher in referral hospitals in Burkina Faso at all pause points excluding immediate post-partum.

For peer review only - http://bmjopen.bmj.com/site/about/guidelines.xhtml

Conclusions: Women delivering in healthcare facilities do not always receive proven EBPs needed to prevent poor childbirth outcomes. There is a need for quality improvement interventions.

Keywords: Quality maternal and newborn care, Burkina Faso, Côte d'Ivoire

Strengths and limitations of the study

- Random selection of health facilities in two different countries and observation of sufficient sample sizes of birth events in each country;
- Direct and prospective assessment of healthcare workers' adherence to essential best practices (EBPs) during and after childbirth by trained healthcare providers as opposed to retrospective ascertainment through medical records in many previous studies
- Use of context-specific validated EBPs to maximise relevance of the study findings
- Each country data are not representative of the whole country
- Because of the direct observation of the practices, we cannot rule out the Hawthorne effect and the data were collected by eight data collectors in each country and are thus subject to an inter-rater reliability bias even though they were trained to standardize the processes.

For peer review only

For peer review only - http://bmjopen.bmj.com/site/about/guidelines.xhtml

INTRODUCTION

Reducing the high burden of preventable maternal and neonatal deaths remains an important health-related goal in the sustainable development era. Although tremendous progress has been achieved globally in this area during the last two decades, most of the highly affected countries have failed to meet the target of reducing their 1990 maternal mortality ratios by 75% by 2015.[1,2] Around 830 women are still dying every day from pregnancy and childbirth-related complications, of which 99% are occurring in low- and middle-income countries and more than 50% in sub-Saharan Africa (SSA).[1,3] There is strong evidence showing that the largest burden of maternal death is clustered around the time of childbirth and immediate post-partum period.[4] A global advocacy for skilled attendance at each birth, aiming to reduce preventable maternal deaths during the Millennium Development Goals (MDGs) era,[5,6] resulted in a significant increase in the access and uptake of childbirth services globally, including in SSA countries.[7,8] This was however not paralleled by the expected reduction in maternal and infant mortality rates in low-resource settings.[9] A skilled birth attendant and health facility delivery will improve maternal and newborn outcomes only if they encompass the implementation of proven effective interventions needed to prevent or manage major causes of maternal and newborn morbidity and mortality.

When the quality of care is substandard, these principles are challenged.[10] The poor quality of care in health facilities, specifically in low-resource settings, represents a neglected agenda[11] and is increasingly recognized as a major contributing factor to persistent high levels of preventable maternal and newborn morbidity and mortality.[10] Closing the gaps in quality of care will be central to the achievement of the global commitment towards Universal Health Coverage (UHC).[12] One of the pillars of the UHC will be to make quality services available to individuals and communities to meet their health-related needs without experiencing financial hardships. Quality of care is however complex and encompasses

For peer review only - http://bmjopen.bmj.com/site/about/guidelines.xhtml

various approaches and dimensions. Quality of maternal and newborn care is very often assessed through delivery outcomes or the availability of resources needed to implement effective interventions,[13–15] rarely using process indicators.[16] While childbirth outcomes are relevant indicators of quality of care, they do not provide information on the process of care and, therefore, neither on quality gaps that need to be filled. Very few studies have investigated the quality of maternal and newborn care in SSA using process indicators. In this study, we intend to assess and compare the quality of intra-partum and immediate post-partum care in two SSA countries using established process indicators.[16]

METHODS

Study settings

Burkina Faso (low-income country) and Côte d'Ivoire (low-middle income country), two SSA countries with maternal mortality ratios among the highest in the world (310 and 614 per 100000 live births respectively),[17,18] have both witnessed significant increase in the uptake of childbirth services during the last two decades, with health facility births reaching nearly 90%.[19] Yet, both countries are struggling to achieve the desire reduction in maternal deaths and the current pace must be further accelerated if they are to meet Target 3.8 of the Sustainable Development Goals of not having more than 140 maternal deaths per 100000 live births by 2030.[20] The study was carried out in two regions where the collaborating research centres have locations, one in each country. In Burkina Faso, the Central-North health region hosted the study, while in Côte d'Ivoire, it was conducted in the Agneby-Tiassa-Mé health region. The two regions were purposively selected based on the presence of a geographic location of the research centers that partner to conduct the study. In each region we randomly drew five from the existing (6 in each country).

Both regions have their capital cities located approximately 100 km away from the respective capital city of its country. The two regions have similar healthcare delivery systems with a regional hospital, followed by district hospitals, each serving a health district comprised of several primary healthcare centres (PHCFs). The central-north health region in Burkina Faso has six health districts and 130 primary healthcare facilities (PHCFs) and the Agnéby-Tiassa-Mé health region of Côte d'Ivoire is divided into seven health districts with 131 PHCFs.

For peer review only
For peer review only - http://bmjopen.bmj.com/site/about/guidelines.xhtml

Study design

We used a facility-based cross-sectional study in government-owned health facilities, enabling a direct and passive observation of the healthcare providers' management of deliveries.

Study population, sampling, and sample size calculation

In each selected region, we randomly selected five health districts. The district hospitals of the selected health districts along with the regional hospital were systematically included in the study in each country. Based on the average childbirth caseloads per level of care (hospital and PHCF) and a maximum length of facility stay of 5 days per data collector's team, it was estimated that a minimum of 65 PHCFs would be needed in each country to achieve the calculated sample size. In the catchment area of each selected health district, up to 15 eligible PHCFs were randomly selected to be included in the study. Eligible PHCFs were those with a minimum average caseload of 1.5 childbirths per day as reported in the health records of the same period in the previous year (during the last quarter of 2017).

The study population comprised

- All the healthcare providers involved in intra-partum and immediate post-partum care, irrespective of their qualifications. They were offered to participate in the study and we excluded healthcare providers who were in the health facility for their qualifying internship.
- All the pregnant women in labour, admitted without c-section or referral needed to be identified at admission. We consecutively recruited all eligible pregnant women during the data collection period in each health facility. Exclusion criteria were being aged <18 years in both countries, pregnant women admitted at an advanced stage of

For peer review only - http://bmjopen.bmj.com/site/about/guidelines.xhtml

the labour (third stage) with frequent (≤5-min apart) and intense uterine contractions that would prevent them from providing valid informed consent.

The sample size of pregnant women was calculated using the conservative proportion of 50% adherence to each essential best practice (EBP) with a precision of 5% and type I error of 5%. To account for the clustering nature of the data, the calculated sample size was adjusted using a design effect based on an intra-class correlation coefficient of 0.01[21] and a size of 15 observations per group. The minimum sample size was estimated to be 425 pregnant women to be included in the observation per pause-point in each country, giving a minimum total number of 1700 of observations per country.

Data collection tools and processes

Data collectors with a medical background (medical students, midwives) and who had undergone a two-day training including in study-room role plays and a field testing of the study tools, were appointed in the delivery room to directly observe and document healthcare providers' adherence to EBPs at four critical points: admission, before the woman starts pushing, immediately post-partum (soon after birth up to ≤ 1h), and before discharge from the health facility. During the field test, a pair of data collectors had to observe at least two pause-points of the same birth event along with a supervisor. Data collectors' measurements were compared within each pair of data collectors and to the measurements of the supervisor. The observed variations were discussed.

The EBPs included in this study were derived from the World Health Organization Safe Childbirth Checklist (WHO-SCC)[22] complemented by a list of other validated quality indicators for SSA[16] (see Table 1). A total of 34 EBPs were observed during each birth event. Adherence to each EBP was measured using a binary yes/no variable. The observation was strictly passive with minimum interaction between observer and healthcare workers and

For peer review only

For peer review only - http://bmjopen.bmj.com/site/about/guidelines.xhtml

patients. The data collectors were only allowed to intervene in the event of a life-threatening complication. The data collection took place from 12 November to 20 December 2018 and lasted for up to five days in each health facility. Observations were conducted every day of the week and at any time without discontinuation, provided an eligible participant was present. A data collector could observe all or part of the four pause-points for each birth event. However, data pertaining to the same critical juncture were fully collected by the same data collector. Thus, once a data collector had initiated the observation for a given pause-point, he/she could get off work only after having filled in all the items pertaining to that pause point.

For peer review only

For peer review only - http://bmjopen.bmj.com/site/about/guidelines.xhtml

Table 1: Essential best practices (EBPs) to be observed for health workers' compliance at each pause-point

N°	Admission	Before pushing	Soon after birth	Before discharge
1	○ Washes his/her hands before examination	○ Sterile gloves available	○ Clamps or ties cord correctly	○ Assesses the mother for vaginal bleeding
2	○ Wears sterile gloves before vaginal examination	○ Soap and clean water available	○ Immediately dries the baby with clean towel	○ Assesses the baby for the quality of breastfeeding
3	○ Asks for vaginal bleedings	○ Prepares a prefilled syringe with uterotonics for AMTSL	○ Assesses for vaginal and perineal lacerations	○ Discusses postpartum family planning with the woman/couple
4	○ Asks for vision blurred	○ Clean towel available	○ Assesses for placenta completeness	○ Plans postpartum visits and makes sure they will consult in the event of a complication
5	○ Checks HIV status	○ Sterile blade or scissor available	○ Assesses new born for need of reference	
6	○ Measures blood pressure	○ Face mask available	○ Takes mother vital signs within 15 minutes after	
7	○ Measures pulse	○ Suction device available	○ Palpates uterus within 15 minutes after birth	
8	○ Uses partograph where indicated	○ Assistant birth attendant identified and ready to intervene-	○ Initiates breastfeeding within 1 hour	
9	○ Measures temperature-yes		○ Skin-to-skin contact initiated within 1 hour	
10	○ Assesses for need of referral		○ Informs companion/woman to call if complications	
11	○ Encourages companion to assist			
12	○ Informs companion/woman to call in case of complications			

For peer review only

For peer review only - http://bmjopen.bmj.com/site/about/guidelines.xhtml

Data analysis

Descriptive statistics were computed using means and standard deviations or medians and interquartile ranges for numerical variables, and proportions were used to describe categorical variables. We calculated the level of adherence to each EBP as the proportion of childbirth events in which the EBP was effectively performed. These proportions were computed along with their 95% confidence intervals (CIs). For each pause-point, based on complete cases, a quality score was computed as the number of EBPs effectively performed at each birth event. We further used the Wilcoxon Ranksum test to compare quality scores at the four clinical junctures between PHCFs and hospitals in each country. Because the study finally included varied numbers of observations per health facility, all the analyses were weighted for the clinic size. A p-value <0.05 indicated statistical significance.

Ethics

The research protocol obtained ethical approvals from the national ethics review committees of Burkina Faso (reference number 2017-4-043) and Côte d'Ivoire (161-18/MSHP/CNE SVS-Kp). It was also approved by the ethics review committee of the WHO (protocol ID ERC.0002951). Written informed consent was obtained from all participants of the study. Data were collected, managed, and analysed in a way to ensure the confidentiality of study participants.

Patient and public involvement

There has been no patient and/or public involvement in the study design, data collection, data analysis, and writing of this research.

For peer review only

For peer review only - http://bmjopen.bmj.com/site/about/guidelines.xhtml

RESULTS

A total of 73 and 69 health facilities in Burkina Faso and Côte d'Ivoire, respectively, were included in the study. In total, 532 and 627 pregnant women in labour gave informed consent to be included in the observations for pause-point I in Burkina Faso and Côte d'Ivoire, respectively. Once included in the observation (pause-point I), a woman and thereafter her newborn were observed through the subsequent pause-points unless they were referred to another health facility or transferred to a different service. Another reason why a mother–newborn pair could not be observed at the fourth pause-point was them still being in the health facility at the time the data collectors had completed their overall stay in that particular facility. The number of birth events observed by pause-point by country and by health facility type are presented in Table 2. As stated above, the reduction in participant numbers from each pause-point to the next was a result of referral, transfer, and not yet being discharged from the health facility at the end of observation period.

Compliance with EBPs at admission

In Burkina Faso, adherence to EBPs ranged from 7.96% (95% CI: 5.66–11.06; wears sterile gloves for vaginal examination) to 99.27% (95% CI: 97.74–99.76; assesses the woman for need of referral). The adherence was significantly higher in referral hospitals as compared to PHCFs for the following EBPs: asks about vaginal bleeding ($p<0.05$), asks about blurred vision ($p<0.01$), measures blood pressure ($p<0.001$), measures pulse ($p<0.01$), and measures temperature ($p<0.05$). Adherence was significantly higher in PHCFs for the EBP 'encourages companion/woman to call in case of complication' ($p<0.01$).

In Côte d'Ivoire, adherence to EBPs ranged from 6.71% (95% CI: 3.94–11.20; washes his/her hands before examination) to 84.97% (95% CI: 81.24–88.09; assesses the woman for need of referral). The compliance was significantly higher in PHCFs than referral hospitals for the

For peer review only - http://bmjopen.bmj.com/site/about/guidelines.xhtml

following EBPs: asks about vaginal bleeding (p<0.01), measures blood pressure (p<0.001), measures pulse (p<0.01), measures temperature (p<0.01), and encourages companion/woman to call in case of complication (p<0.001).

Compliance with EBPs at the immediate pre-pushing phase

In Burkina Faso, all EBPs were implemented in more than half of the deliveries that took place in referral hospitals. The same was observed in PHCFs, except for availability of bag and mask, which was observed in 39.34% (95% CI: 34.38–44.53) of the deliveries. The adherence was significantly higher in referral hospitals than PHCFs for three EBPs: prepares pre-filled syringe with uterotonics for active management of the third stage of labour (AMTSL) (p<0.05), bag and mask available (p<0.001), and assistant birth attendant ready to intervene if needed (p<0.001).

In Côte d'Ivoire, except for availability of bag and mask and availability of suction device, all other EBPs were adhered to in more than half of the deliveries in both types of health facilities. Bag and mask were available in 5.67% (95% CI: 3.68–8.77) and 18.74% (95% CI: 12.17–27.69) of the deliveries in PHCFs and referral hospitals, respectively. Availability of suction device (p<0.001) and sterile blade or scissor (p<0.01) were also better in referral hospitals than PHCFs and preparation of a pre-filled syringe with uterotonics for AMTSL was best adhered to in PHCFs (p<0.001).

Compliance to EBPs immediately post-partum

In Burkina Faso, adherence was above 50% for all EBPs ranging from 51.86% (95% CI: 46.58–57.10; takes vital signs within 15 min) to 99.71% (95% CI: 98.67 to EBPs 99.94; assesses the newborn for need of referral). Significantly higher adherence was observed in referral hospitals regarding encouraging companion/woman to call in case of complication (p<0.01).

For peer review only

For peer review only - http://bmjopen.bmj.com/site/about/guidelines.xhtml

264 In Côte d'Ivoire, adherence ranged from 19.66% (95% CI: 15.49–24.61; clamps or ties cord correctly) to 98.5% (95% CI: 96.91–99.28; assesses for vaginal or perineal lacerations). The adherence was significantly higher in PHCFs for the following EBPs: Assesses for vaginal and perineal lacerations ($p<0.01$), assesses newborn for need of referral ($p<0.01$), palpates uterus within 15 min after birth ($p<0.05$), initiates breastfeeding within 1 h ($p<0.01$), and skin-to-skin contact initiated within 1 h ($p<0.001$).

Compliance to EBPs before discharge from health facility

When leaving the facility, 47.14% (95% CI: 37.09–47.36) of women in Burkina Faso were offered a discussion on post-partum family planning with a significantly higher performance in referral hospitals ($p<0.001$). In Cote d'Ivoire, the quality of breastfeeding was assessed in 26.48% (95% CI: 21.46–32.19) cases and was best adhered to in PHCFs ($p<0.001$).

The levels of adherence to all EBPs per pause-point, country, and health facility type are presented in Table 2.

For peer review only

For peer review only - http://bmjopen.bmj.com/site/about/guidelines.xhtml

Table 2: Compliance to essential best practices by pause-point and by health facility type in Burkina Faso and Côte d'Ivoire.

**Essential best practices**	**Burkina Faso**				**Côte d'Ivoire**			
	PHCF		RH		PHCF		RH	
	n/N	% (95% CI)	n/N	% (95% CI)	n /N	% (95% CI)	n/N	% (95% CI)
**Admission**	**428**		**104**		**513**		**114**	
Washes his/her hands before examination	86/420	20.50 (16.72-24.86)	20/101	19.02(12.48-27.89)	29/501	06.72(03.94-11.24)	5/114	05.00(02.10-11.45)
Wears sterile gloves before vaginal examination	33/425	07.94(05.64-11.07)	11/103	10.73(06.03-18.39)	49/500	07.03(05.23-09.39)	6/114	05.60(02.52-11.96)
Asks for vaginal bleedings	181/422	**41.62(36.57-46.84)**	55/104	**54.18(44.46-63.58)**	144/504	**33.97(28.96-39.38)**	23/114	**20.40(13.90-28.92)**
Asks for vision blurred	160/422	**37.04(32.12-42.24)**	53/104	**52.51(42.82-62.01)**	122/504	27.67(23.29-32.52)	28/114	25.40(18.12-34.38)
Checks HIV status	333/425	79.25(75.00-82.94)	89/104	86.62(78.85-91.83)	344/497	66.08(60.69-71.08)	73/113	65.24(55.94-73.51)
Measures blood pressure	305/428	**72.32(67.48-76.68)**	94/103	**90.95(83.45-95.25)**	414/509	**81.21(76.66-85.04)**	70/114	**63.63(54.42-71.94)**
Measures pulse	170/428	**39.87(34.85-45.10)**	54/102	**54.36(44.55-63.85)**	230/506	**48.57(43.18-54.00)**	35/114	**33.56(25.25-43.02)**
Uses partograph where indicated[1]	225/428	53.11(47.90-58.25)	53/104	50.36(40.73-59.97)	90/508	17.72(14.04-22.13)	24/114	23.80(16.57-32.94)
Measures temperature	277/428	**65.64(60.50-70.43)**	79/104	**76.61(67.49-83.79)**	207/513	**42.90(37.60-48.36)**	29/114	**27.47(18.83-36.70)**
Assesses for need of referral	425/428	99.26(97.74-99.76)	104/104	100	441/512	**85.04(81.27-88.16)**	89/114	**76.6(67.65-83.67)**
Encourages companion to assist to the childbirth	176/422	**41.47(36.49-46.63)**	26/103	**24.59(17.22-33.82)**	43/474	08.64(06.22-11.88)	11/109	11.14(06.27-19.03)
Companion/woman informed to call if complication	326/424	86.15(82.06-89.42)	101/104	96.7(90.07-98.96)	277/472	**66.07(60.93-70.86)**	45/105	**47.56(38.03-57.26)**
**Before pushing or before c-section**	**416**		**99**		**477**		**109**	
Sterile gloves available	405/416	97.65(95.78-98.71)	96/99	96.70(90.13-98.94)	458/477	93.21(87.48-96.43)	104/109	95.54(89.61-98.16)
Soap and clean water available	259/416	63.42(58.23-68.31)	68/99	70.37(60.73-78.49)	365/477	71.15(65.60-76.12)	83/109	75.65(66.56-82.91)
Prepares a prefilled syringe with uterotonics for AMTSL	348/416	**83.19(79.05-86.65)**	92/99	**92.85(85.71-96.56)**	372/477	**83.74(80.22-86.74)**	69/109	**68.05(58.93-75.96)**
Clean towel available	389/416	94.41(91.93-96.17)	96/99	96.90(90.80-99.00)	416/477	85.97(82.23-89.02)	102/109	92.92(85.87-96.60)
Sterile blade or scissor available	400/416	96.56(94.42-97.90)	98/99	99.00(93.21-99.86)	374/477	**76.15(70.79-80.79)**	98/109	**88.55(80.54-93.53)**
Bag and mask available	168/416	**39.15(34.16-44.37)**	76/99	**76.12(66.58-83.60)**	22/477	**05.67(03.63-08.77)**	18/109	**18.73(12.17-27.69)**

[1] Per national guidelines of both countries, a partograph is indicated at admission only if cervix≥4cm

For peer review only

For peer review only - http://bmjopen.bmj.com/site/about/guidelines.xhtml

	Burkina Faso				Côte d'Ivoire			
Suction device available	312/416	74.57(69.54-79.02)	80/99	80.17(70.96-86.99)	99/477	**17.89(14.57-21.76)**	60/109	**58.06(48.55-67.00)**
Assistant birth attendant ready to intervene	206/416	**49.97(44.69-55.24)**	76/99	**76.13(66.58-83.62)**	415/476	84.87(80.55-88.37)	95/109	88.14(80.86-92.89)
**Soon after birth (within one hour)**	**415**		**99**		**477**		**109**	
Clamps or ties cord correctly	260/415	61.72(56.40-66.77)	56/99	58.42(48.45-67.74)	82/477	**19.57(15.33-24.57)**	32/109	**32.46(24.08-42.14)**
Immediately dries the baby with clean towel	412/415	99.23(97.60-99.75)	99/99	100	441/477	92.37(89.52-94.49)	103/109	93.96(87.18-97.27)
Assesses for vaginal and perineal lacerations	393/415	94.81(92.11-96.63)	95/99	96.18(90.23-98.57)	470/477	**99.54(96.91-99.31)**	102/109	**93.13(86.24-96.70)**
Assesses for placenta completeness	235/414	55.17(49.83-60.40)	45/99	47.42(37.65-57.40)	171/476	37.02(32.05-42.28)	33/109	33.18(24.74-42.86)
Assesses new-born for need of reference	413/415	99.71(98.65-99.94)	98/99	99.00(93.21-99.86)	418/477	**86.24(82.47-89.30)**	82/108	**74.46(65.12-81.99)**
Takes mother vital signs within 15 minutes after	208/415	51.82(46.52-57.09)	60/99	58.38(48.26-67.85)	234/477	47.01(41.69-52.40)	38/108	38.44(29.52-48.21)
Palpates uterus within 15 minutes after birth	337/415	83.78(79.75-87.14)	79/99	79.44(70.16-86.39)	289/477	**59.11(53.44-64.55)**	45/108	**44.80(35.61-54.53)**
Initiates breastfeeding within 1 hour	298/395	75.85(71.01-80.06)	63/93	67.36(57.08-76.20)	184/434	**48.24(42.46-54.06)**	28/100	**30.14(21.72-40.16)**
Skin-to-skin contact initiated within 1 hour	331/406	81.81(76.98-85-81)	74/97	76.02(66.43-83.56)	275/465	**62.14(56.94-67.09)**	34/103	**35.09(26.29-45.03)**
Informs companion/woman to call if complications	350/415	**85.15(80.92-88.57)**	95/99	**95.80(89.20-98.44)**	365/474	78.03(73.47-82.00)	74/105	70.86(61.39-78.81)
**Before discharge from the health facility**	**411**		**93**		**474**		**106**	
Assesses the mother for vaginal bleeding	291/408	70.82(65.54-75.59)	73/93	79.82(70.59-86.69)	310/474	68.68(63.46-73.46)	64/105	64.62(55.10-73.10)
Assesses the baby for the quality of breastfeeding	227/408	55.56(50.16-60.84)	49/92	52.46(42.14-62.59)	129/474	**26.60(21.55-32.35)**	6/106	**06.43(02.92-13.58)**
Discusses postpartum family planning with the woman/couple	173/409	**42.01(36.94-47.26)**	64/92	**67.24(56.67-76.31)**	169/473	29.10(24.52-34.12)	35/106	30.19(22.27-39.50)
Plans postpartum visits and informs to consult if complications	349/409	**84.28(79.20-88.30)**	86/92	**93.21(85.60-96.95)**	380/474	**77.68(72.94-81.81)**	73/106	**67.85(58.20-76.18)**

PHCF: Primary Health Care Facility; RH: Referral Hospital

For peer review only - http://bmjopen.bmj.com/site/about/guidelines.xhtml

The analysis showed no significant variation between night and day shifts, nor there was a difference between weekdays and weekends in adherence to EBPs at any pause-point.

The overall mean quality scores were consistently higher in referral hospitals than PHCFs in Burkina Faso at all pause-points ($p<0.01$) except immediately post-partum when there was no significant difference. In Côte d'Ivoire, the overall quality score was better in PHCFs at admission ($p<0.001$), immediately post-partum ($p<0.001$), and before discharge from the health facility ($p<0.01$). It was higher in referral hospitals at the immediate pre-pushing phase ($p<0.01$). The distribution of the quality scores by pause-point and by country is depicted in Figure 1.

For peer review only

DISCUSSION

In this study, we assessed healthcare providers' adherence to EBPs in delivery care using direct observation. Our results showed a wide diversity in adherence to EBPs ranging from very low to very high. Overall, adherence to EBPs tended to be better in referral hospitals as compared to PHCFs in Burkina Faso. In Côte d'Ivoire, the compliance was higher in PHCFs than referral hospitals. Important quality gaps were shown in both countries. Hand hygiene (washing hands before examination and wearing sterile gloves for each vaginal examination) practices were particularly very poor in both countries despite the widespread availability of hand hygiene facilities in both countries (hand washing facilities with soap and clean water were found in 94.74% and 74.96% of the maternity wards of surveyed healthcare facilities in Burkina Faso and Côte d'Ivoire, respectively). With the equipment available, adherence to hygiene routines is a fundamental and non-demanding practice for prevention of infections.[23,24] Yet, non-compliance is widespread and remains a source of concern globally.[25,26]

History-taking of critical information such as vaginal bleeding or blurred vision during pregnancy was sub-optimal in Burkina Faso and poor in Côte d'Ivoire. Omission of blood

For peer review only - http://bmjopen.bmj.com/site/about/guidelines.xhtml

311 pressure measurement was common, and occurred in one out of four women in Côte d'Ivoire.
312 Temperature was not measured in two-thirds of the women in labour, missing an opportunity
313 to identify ongoing episodes of infection.

314 These gaps in initial assessment can cause at-risk patients or prevailing complications to go
315 undetected. It is also noteworthy that, despite being an ancient and long-systematized
316 intervention, a partograph to monitor labour was used during less than half of the deliveries in
317 both countries. The low utilization of the partograph and its poor quality when used are well
318 recognized and have been widely reported in the literature.[27–29]

319 Assessment of vaginal bleeding and perineal lacerations and preparation of a prefilled syringe
320 with uterotonics for AMTSL were satisfactorily adhered to in both countries (>75%). These
321 are important practices for the prevention, early detection, and management of post-partum
322 haemorrhage (PPH), a leading cause of maternal death.[4] Bedside availability of prepared
323 oxytocin improves its timely administration.[30] Increased compliance to these standards may
324 be the result of rising awareness of the importance of PPH as a leading cause of death and the
325 emphasis put on its prevention and management in both initial and refresher trainings for
326 healthcare providers.

327 Implementation of other practices equally relevant to PPH: the performance of 'assessment of
328 the completeness of the placenta' and 'monitoring of maternal vital signs' was however very
329 low, and only complied with for around half of the women in Burkina Faso for both EBPs,
330 and to one-third and three-sevenths of the women, respectively, in Côte d'Ivoire. These are
331 life-saving low-cost practices, but require health-worker awareness and knowledge on the
332 purpose and rationale for providing these services, in addition to clinical skills in executing
333 them. The low proportion of women in both countries that received these interventions
334 represent unmet needs of urgent action to address these cost-effective practices. Our findings
335 also highlight the complexity of the peri- and immediate post-partum care, wherein a series of

For peer review only - http://bmjopen.bmj.com/site/about/guidelines.xhtml

equally important steps need to be taken to minimize risks of maternal morbidity, and how failure to address all of these puts women at risk of severe consequences.

Thermal management of newborns was satisfactorily adhered to in both countries through the availability of clean towels before the woman started pushing and drying the newborn immediately post-partum. Compliance with other lifesaving interventions for newborns (skin-to-skin contact, early initiation of breastfeeding) was beyond 75% women in Burkina Faso and barely half of the women in Côte d'Ivoire.

Overall, supportive services (encouraging companion to assist in the delivery, identifying and preparing an assistant birth attendant ready to help) at any point in time tend to be less adhered to as compared to other standards of care.

The health facilities in both countries rarely used the opportunity to provide post-partum contraceptive advice to the patients. The same has been previously reported[21] and is suggestive of health workers' propensity to give precedence to interventions needed to prevent or treat imminent and tangible complications. Given the large unmet needs of modern contraceptives in both countries, implementation of post-partum family planning programmes is an important strategy to close the gap. Knowledge translation and follow-up of patients is needed between healthcare workers engaged in intra-partum care and family planning.

Noticeably, a significant proportion of women and or companions received information related to potential complications and signs of danger and what to do in case they occur in both countries from admission to discharge. Apart from the immediate post-partum period wherein there was no significant difference, compliance with standards in Burkina Faso tended to be better at all junctures in referral hospitals than PHCFs. Such a systematic difference was not observed in Côte d'Ivoire. This difference may be because in Burkina Faso, there is a wider existence of categories of auxiliary nurses and auxiliary midwives who

For peer review only

For peer review only - http://bmjopen.bmj.com/site/about/guidelines.xhtml

predominantly work in PHCFs, while in Côte d'Ivoire, deliveries are attended to by nurses and midwives at PHCFs as well as referral hospitals.

Study limitations

In interpreting our results, some limitations should be considered. We carried out a direct observation of facility-based deliveries in a sample of health facilities in two selected regions and the findings may not be necessarily representative of each individual country. Although we did not provide information to healthcare providers on the items being observed, we cannot rule out the Hawthorne effect and some may have changed their healthcare practices during the study period. The data collection was carried out by eight data collectors in each country. Although they were trained by the same research team to standardize processes and judgements, we cannot rule out issues related to inter-rater reliability. Further analysis including the level of adherence to EBPs according to providers qualification and working experience would have added value to this work. Finally, the non-compliance to a given standard does not always imply that the observed healthcare provider failed to do so. In some cases, the supplies needed to administer the intervention may have been unavailable.

For peer review only

CONCLUSION

Overall, compliance to EBPs during delivery was insufficient and important gaps in quality of care were identified. A significant number of women in Burkina Faso and Cote d'Ivoire missed out on important interventions, especially those related to infection prevention and control and prevention of PPH. In addition, there were lost opportunities in relation to implementation of post-partum contraceptive strategies. Conditional on the availability of supplies, the investigated EBPs were relatively low cost and easy to implement. Quality-improvement interventions in the form of reminder tools for healthcare providers, such as the

For peer review only - http://bmjopen.bmj.com/site/about/guidelines.xhtml

WHO-SSC have the potential to sustain currently performed best practices while filling the gaps that persist.

Authors' contributions

All authors meet the criteria recommended by the International Committee of Medical Journal Editors (ICMJE). All authors made substantial contributions to conception and design, acquisition of data or analysis and interpretation of data. KS,MT,AYML conceived the study design. MT analysed the data and proposed the first draft of the paper. KS, AYML, KRK, YWME, TKA, SFB, SCA and TA contributed to interpret the data and have revised and contributed to the successive drafts. The final version of the paper was revised and approved by all the authors.

Acknowledgment

We are grateful to WHO Human Reproduction Program (WHO/HRP) for funding the research.

Data availability statement

The data presented in this study are from the Checklist study conducted in Burkina Faso and Côte d'Ivoire. Data can be accessed upon request sent to the principal investigator of the study, Prof Seni Kouanda at senikouanda@gmail.com. Access will be granted only after careful and due consideration of the compliance with the ethics requirements and the data policy of the Institut de Recherche en Sciences de la Santé.

Funding

This work was supported by WHO/HRP. The funders have no role in the design of the research, nor they have in the collection, analysis, and interpretation of the data.

Competing interests

All authors declare that they have no competing interests.

For peer review only

For peer review only - http://bmjopen.bmj.com/site/about/guidelines.xhtml

REFERENCES

1. Alkema L, Chou D, Hogan D, Zhang S, Moller A-B, Gemmill A, Fat DM, Boerma T, Temmerman M, Mathers C, Say L. Global, regional, and national levels and trends in maternal mortality between 1990 and 2015, with scenario-based projections to 2030: a systematic analysis by the UN Maternal Mortality Estimation Inter-Agency Group. The Lancet. 2016 Jan;387(10017):462–74.

2. Victora CG, Requejo JH, Barros AJD, Berman P, Bhutta Z, Boerma T, Chopra M, de Francisco A, Daelmans B, Hazel E, Lawn J, Maliqi B, Newby H, Bryce J. Countdown to 2015: a decade of tracking progress for maternal, newborn, and child survival. The Lancet. 2016 May;387(10032):2049–59.

3. World Health Organization, UNICEF, United Nations, Department of Economic and Social Affairs, Population Division, World Bank. Trends in maternal mortality: 1990 to 2015 : estimates by WHO, UNICEF, UNFPA, World Bank Group and the United Nations Population Division [Internet]. 2015 [cited 2017 Dec 17]. Available from: http://www.who.int/reproductivehealth/publications/monitoring/maternal-mortality-2015/en/

4. Ronsmans C, Graham WJ, Lancet Maternal Survival Series steering group. Maternal mortality: who, when, where, and why. Lancet. 2006 Sep 30;368(9542):1189–200.

5. Bullough C, Meda N, Makowiecka K, Ronsmans C, Achadi EL, Hussein J. REVIEW: Current strategies for the reduction of maternal mortality: REVIEW. BJOG Int J Obstet Gynaecol. 2005 Jun 29;112(9):1180–8.

6. World Health Organization. Making pregnancy safer the critical role of the skilled attendant: a joint statement by WHO, ICM and FIGO. Geneva: World health organization; 2004.

7. Udo IE, Doctor HV. Trends in Health Facility Births in sub-Saharan Africa: An Analysis of Lessons Learned under the Millennium Development Goal Framework. Afr J Reprod Health [Internet]. 2017 May 22 [cited 2017 Dec 18];20(3). Available from: https://www.ajrh.info/index.php/ajrh/article/view/175

8. Doctor HV, Nkhana-Salimu S, Abdulsalam-Anibilowo M. Health facility delivery in sub-Saharan Africa: successes, challenges, and implications for the 2030 development agenda. BMC Public Health. 2018 Dec;18(1):765.

9. Ekirapa-Kiracho E, Waiswa P, Rahman MH, Makumbi F, Kiwanuka N, Okui O, Rutebemberwa E, Bua J, Mutebi A, Nalwadda G, Serwadda D, Pariyo GW, Peters DH. Increasing access to institutional deliveries using demand and supply side incentives: early results from a quasi-experimental study. BMC Int Health Hum Rights. 2011;11 Suppl 1:S11.

10. Kongnyuy EJ, Mlava G, van den Broek N. Facility-Based Maternal Death Review In Three Districts In The Central Region of Malawi. Womens Health Issues. 2009 Jan;19(1):14–20.

11. van den Broek N, Graham W. Quality of care for maternal and newborn health: the neglected agenda: Quality of care for maternal and newborn health. BJOG Int J Obstet Gynaecol. 2009 Oct;116:18–21.

12. Chan M, Director-General W. QUALITY OF CARE IN THE CONTEXT OF UNIVERSAL HEALTH COVERAGE (UHC). :2.

13. Admasu K, Haile-Mariam A, Bailey P. Indicators for availability, utilization, and quality of emergency obstetric care in Ethiopia, 2008. Int J Gynecol Obstet. 2011 Oct;115(1):101–5.

14. Wilunda C, Oyerinde K, Putoto G, Lochoro P, Dall'Oglio G, Manenti F, Segafredo G, Atzori A, Criel B, Panza A, Quaglio G. Availability, utilisation and quality of maternal and neonatal health care services in Karamoja region, Uganda: a health facility-based survey. Reprod Health [Internet]. 2015 Dec [cited 2017 Dec 18];12(1). Available from:

For peer review only - http://bmjopen.bmj.com/site/about/guidelines.xhtml

http://reproductive-health-journal.biomedcentral.com/articles/10.1186/s12978-015-0018-7
15. Baguiya A, Meda IB, Millogo T, Kourouma M, Mouniri H, Kouanda S. Availability and utilization of obstetric and newborn care in Guinea: A national needs assessment. Int J Gynaecol Obstet Off Organ Int Fed Gynaecol Obstet. 2016 Nov;135 Suppl 1:S2–6.
16. Tripathi V, Stanton C, Strobino D, Bartlett L. Development and Validation of an Index to Measure the Quality of Facility-Based Labor and Delivery Care Processes in Sub-Saharan Africa. Duan T, editor. PLOS ONE. 2015 Jun 24;10(6):e0129491.
17. Institut National de la Statistique et de la Démographie (INSD), ICF International. Enquête Démographique et de Santé et à Indicateurs Multiples du Burkina Faso 2010. Calverton, Maryland, USA; 2012.
18. Institut National de la Statistique (INS), ICF International. Enquête Démographique et de Santé et à Indicateurs Multiples de Côte d'Ivoire 2011-2012. Calverton, Maryland, USA : INSD et ORC Macro.; 2012.
19. Ministère de la Santé. Annuaire Statistique 2015 [Internet]. Ouagadougou; 2016 Mar. Available from: http://www.sante.gov.bf/index.php?option=com_edocman&task=document.viewdoc&id=113&Itemid=1123
20. Alkema L, Chou D, Hogan D, Zhang S, Moller A-B, Gemmill A, Fat DM, Boerma T, Temmerman M, Mathers C, Say L. Global, regional, and national levels and trends in maternal mortality between 1990 and 2015, with scenario-based projections to 2030: a systematic analysis by the UN Maternal Mortality Estimation Inter-Agency Group. The Lancet. 2016 Jan;387(10017):462–74.
21. Kumar S, Yadav V, Balasubramaniam S, Jain Y, Joshi CS, Saran K, Sood B. Effectiveness of the WHO SCC on improving adherence to essential practices during childbirth, in resource constrained settings. BMC Pregnancy Childbirth [Internet]. 2016 Dec [cited 2017 Jan 17];16(1). Available from: http://bmcpregnancychildbirth.biomedcentral.com/articles/10.1186/s12884-016-1139-x
22. Spector JM, Lashoher A, Agrawal P, Lemer C, Dziekan G, Bahl R, Mathai M, Merialdi M, Berry W, Gawande AA. Designing the WHO Safe Childbirth Checklist program to improve quality of care at childbirth. Int J Gynecol Obstet. 2013 Aug;122(2):164–8.
23. Allegranzi B, Pittet D. Role of hand hygiene in healthcare-associated infection prevention. J Hosp Infect. 2009 Dec;73(4):305–15.
24. Pittet D, Allegranzi B, Storr J. The WHO Clean Care is Safer Care programme: Field-testing to enhance sustainability and spread of hand hygiene improvements. J Infect Public Health. 2008;1(1):4–10.
25. Musuuza JS, Barker A, Ngam C, Vellardita L, Safdar N. Assessment of Fidelity in Interventions to Improve Hand Hygiene of Healthcare Workers: A Systematic Review. Infect Control Hosp Epidemiol. 2016 May;37(5):567–75.
26. Anargh V, Singh H, Kulkarni A, Kotwal A, Mahen A. Hand hygiene practices among health care workers (HCWs) in a tertiary care facility in Pune. Med J Armed Forces India. 2013 Jan;69(1):54–6.
27. Asibong U, Okokon IB, Agan TU, Oku A, Opiah M, Essien EJ, Monjok E. The use of the partograph in labor monitoring: a cross-sectional study among obstetric caregivers in General Hospital, Calabar, Cross River State, Nigeria. Int J Womens Health. 2014;6:873–80.
28. Opiah MM, Ofi AB, Essien EJ, Monjok E. Knowledge and Utilization of the Partograph among Midwives in the Niger Delta Region of Nigeria. Afr J Reprod Health Rev Afr Santé Reprod. 2012;16(1):125–32.
29. Mandiwa C, Zamawe C. Documentation of the partograph in assessing the progress of labour by health care providers in Malawi's South-West zone. Reprod Health. 2017 Dec;14(1):134.

For peer review only

For peer review only - http://bmjopen.bmj.com/site/about/guidelines.xhtml

30. Moucheraud C, Gass J, Lipsitz S, Spector J, Agrawal P, Hirschhorn LR, Gawande A, Kodkany B. Bedside Availability of Prepared Oxytocin and Rapid Administration After Delivery to Prevent Postpartum Hemorrhage: An Observational Study in Karnataka, India. Glob Health Sci Pract. 2015 Jun;3(2):300–4.

For peer review only

For peer review only - http://bmjopen.bmj.com/site/about/guidelines.xhtml

Figure legends

Figure 1: The boxplots depict the overall scores of quality of care computed for each pause-point by health facility type and by country. The maximum quality score for any pause-point is equal to the number of essential best practices observed at that pause-point.

For peer review only

For peer review only - http://bmjopen.bmj.com/site/about/guidelines.xhtml

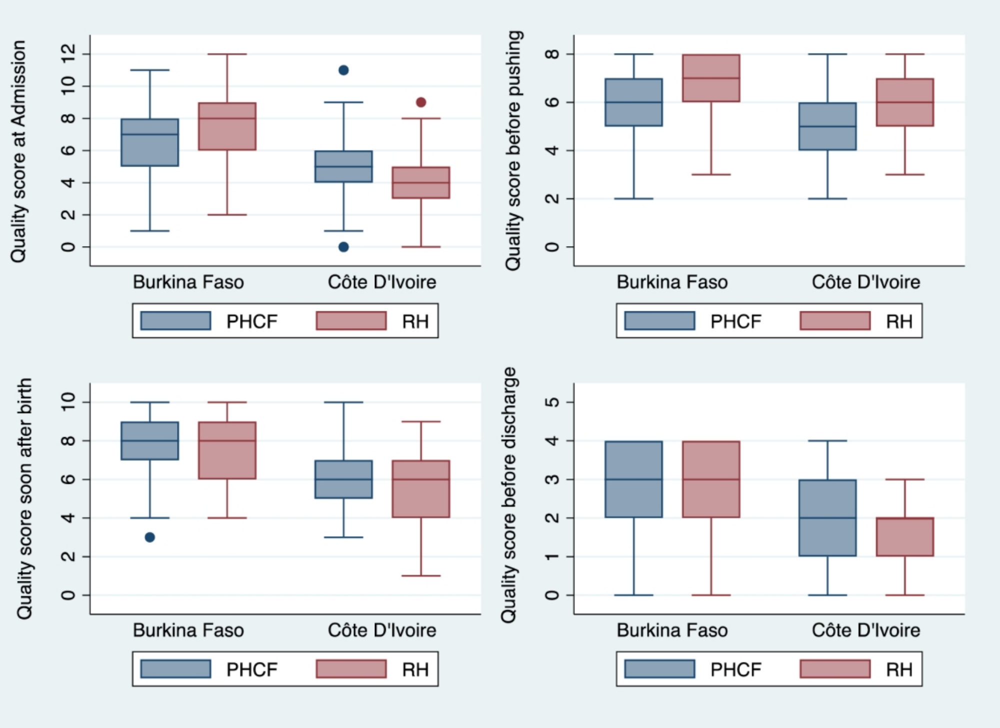

Figure 1: Distribution of quality scores per country and pause-points.

Figure legends

Figure 1: The boxplots depict the overall scores of quality of care computed for each pause-point by health facility type and by country. The maximum quality score for any pause-point is equal to the number of essential best practices observed at that pause-point.

The boxplots depict the overall scores of quality of care computed for each pause-point by health facility type and by country. The maximum quality score for any pause-point is equal to the number of essential best practices observed at that pause-point.

165x168mm (300 x 300 DPI)

STROBE Statement—Checklist of items that should be included in reports of ***cross-sectional studies***

	Item No	Recommendation	Page No
**Title and abstract**	1	(*a*) Indicate the study's design with a commonly used term in the title or the abstract	1 &2
		(*b*) Provide in the abstract an informative and balanced summary of what was done and what was found	2
**Introduction**			
Background/rationale	2	Explain the scientific background and rationale for the investigation being reported	4
Objectives	3	State specific objectives, including any prespecified hypotheses	4
**Methods**			
Study design	4	Present key elements of study design early in the paper	5
Setting	5	Describe the setting, locations, and relevant dates, including periods of recruitment, exposure, follow-up, and data collection	5, 7
Participants	6	(*a*) Give the eligibility criteria, and the sources and methods of selection of participants	6
Variables	7	Clearly define all outcomes, exposures, predictors, potential confounders, and effect modifiers. Give diagnostic criteria, if applicable	8
Data sources/ measurement	8*	For each variable of interest, give sources of data and details of methods of assessment (measurement). Describe comparability of assessment methods if there is more than one group	*7*
Bias	9	Describe any efforts to address potential sources of bias	7
Study size	10	Explain how the study size was arrived at	6 &7
Quantitative variables	11	Explain how quantitative variables were handled in the analyses. If applicable, describe which groupings were chosen and why	9
Statistical methods	12	(*a*) Describe all statistical methods, including those used to control for confounding	9
		(*b*) Describe any methods used to examine subgroups and interactions	NA
		(*c*) Explain how missing data were addressed	9
		(*d*) If applicable, describe analytical methods taking account of sampling strategy	
		(*e*) Describe any sensitivity analyses	
**Results**			
Participants	13*	(a) Report numbers of individuals at each stage of study—eg numbers potentially eligible, examined for eligibility, confirmed eligible, included in the study, completing follow-up, and analysed	9&10
		(b) Give reasons for non-participation at each stage	10
		(c) Consider use of a flow diagram	
Descriptive data	14*	(a) Give characteristics of study participants (eg demographic, clinical, social) and information on exposures and potential confounders	10
		(b) Indicate number of participants with missing data for each variable of interest	
Outcome data	15*	Report numbers of outcome events or summary measures	10 & 11

For peer review only

Main results	16	(*a*) Give unadjusted estimates and, if applicable, confounder-adjusted estimates and their precision (eg, 95% confidence interval). Make clear which confounders were adjusted for and why they were included	12
		(*b*) Report category boundaries when continuous variables were categorized	
		(*c*) If relevant, consider translating estimates of relative risk into absolute risk for a meaningful time period	
Other analyses	17	Report other analyses done—eg analyses of subgroups and interactions, and sensitivity analyses	
**Discussion**			
Key results	18	Summarise key results with reference to study objectives	12
Limitations	19	Discuss limitations of the study, taking into account sources of potential bias or imprecision. Discuss both direction and magnitude of any potential bias	14
Interpretation	20	Give a cautious overall interpretation of results considering objectives, limitations, multiplicity of analyses, results from similar studies, and other relevant evidence	12 & 13
Generalisability	21	Discuss the generalisability (external validity) of the study results	14
**Other information**			
Funding	22	Give the source of funding and the role of the funders for the present study and, if applicable, for the original study on which the present article is based	15

*Give information separately for exposed and unexposed groups.

Note: An Explanation and Elaboration article discusses each checklist item and gives methodological background and published examples of transparent reporting. The STROBE checklist is best used in conjunction with this article (freely available on the Web sites of PLoS Medicine at http://www.plosmedicine.org/, Annals of Internal Medicine at http://www.annals.org/, and Epidemiology at http://www.epidem.com/). Information on the STROBE Initiative is available at www.strobe-statement.org.

For peer review only
